# Structural evolution of fibril polymorphs during amyloid assembly

**DOI:** 10.1016/j.cell.2023.11.025

**Published:** 2023-12-21

**Authors:** Martin Wilkinson, Yong Xu, Dev Thacker, Alexander I.P. Taylor, Declan G. Fisher, Rodrigo U. Gallardo, Sheena E. Radford, Neil A. Ranson

**Affiliations:** 1 Astbury Centre for Structural Molecular Biology, School of Molecular & Cellular Biology, Faculty of Biological Sciences, University of Leeds, Leeds LS2 9JT, UK

## Abstract

Cryoelectron microscopy (cryo-EM) has provided unprecedented insights into amyloid fibril structures, including those associated with disease. However, these structures represent the endpoints of long assembly processes, and their relationship to fibrils formed early in assembly is unknown. Consequently, whether different fibril architectures, with potentially different pathological properties, form during assembly remains unknown. Here, we used cryo-EM to determine structures of amyloid fibrils at different times during *in vitro* fibrillation of a disease-related variant of human islet amyloid polypeptide (IAPP-S20G). Strikingly, the fibrils formed in the lag, growth, and plateau phases have different structures, with new forms appearing and others disappearing as fibrillation proceeds. A time course with wild-type hIAPP also shows fibrils changing with time, suggesting that this is a general property of IAPP amyloid assembly. The observation of transiently populated fibril structures has implications for understanding amyloid assembly mechanisms with potential new insights into amyloid progression in disease.

## Graphical abstract

**Graphical abstract:**
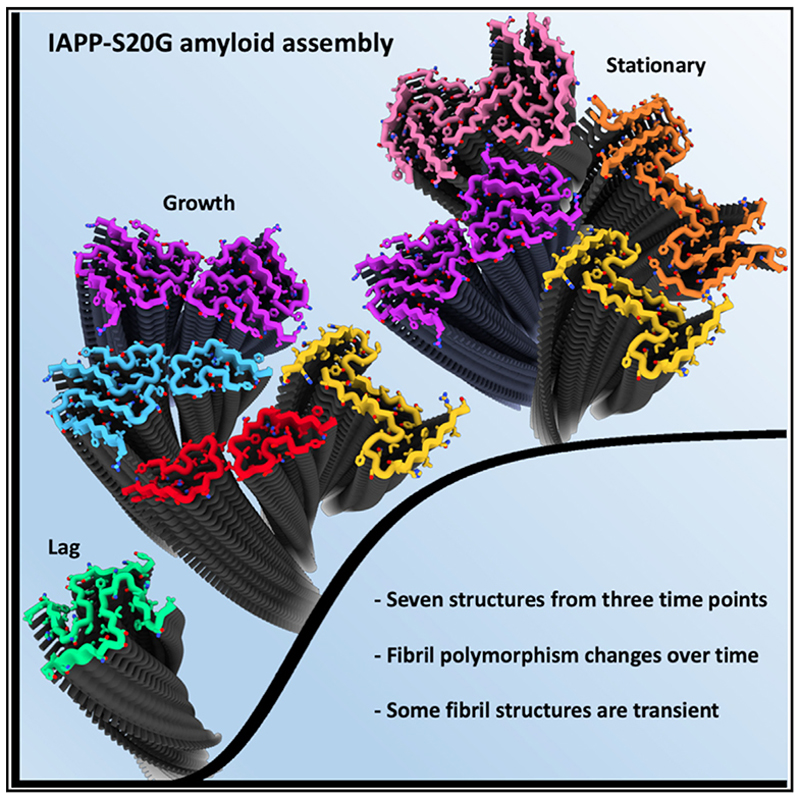

## Introduction

The formation of amyloid fibrils has been described as “the dark side of protein folding”^1^ after genome-wide sequence analysis revealed that most proteins contain self-complementary segments that could assemble into amyloid fibrils.^2^ Although functional amyloids exist (reviewed in Sergeeva and Galkin^3^), amyloid fibril formation is associated with numerous human diseases, including neurodegenerative disorders (Alzheimer and Parkinson’s diseases), amyotrophic lateral sclerosis (ALS), and type 2 diabetes (T2D) (reviewed in Iadanza et al.^4^). Amyloid forms via a nucleation-growth mechanism^5^ that is characterized by lag, growth, and plateau phases and gives rise to sigmoidal fibril self-assembly curves as measured by dyes such as thioflavin T (ThT).^6^ Models have been developed that enable the kinetic processes that generate amyloid fibrils to be described.^7,8^ However, the structural mechanism(s) by which fibrils form remain largely unknown, other than the need for self-associating “steric zipper” β strands that stack into cross-β fibrillar structures.^9–11^ In the past 5 years, a profusion of high-resolution amyloid fibril structures has been determined using cryoelectron microscopy (cryo-EM). These studies show that a diverse array of amyloid structures can be built from the canonical cross-β amyloid fold,^12,13^ including disease-relevant fibril forms.^12–20^ Rather than one polypeptide sequence leading to one amyloid fibril structure, it is now clear that most (if not all) amyloid-forming proteins can generate multiple fibril forms or polymorphs, highlighting that amyloid assembly occurs via a complex cascade of molecular events on a rugged energy landscape.^13,14,18,21–25^

Fibril polymorphism can occur by changes in the number and/or orientation of the same subunit fold or by formation of new subunit folds. Furthermore, changes in reaction conditions or cellular environment and alterations in sequence (e.g., single-point mutations and post-translational modifications) can also result in new polymorphs.^15,26–34^ These structural insights raise important questions about how sequence and solution conditions affect assembly and how the kinetic mechanism of fibril formation (e.g., primary or secondary nucleation) influences the polymorph(s) generated. Understanding why the fibril structures formed *in vitro* are often different to those observed *ex vivo* also remains a major challenge,^15,16^ with a single example (Tau) showing that the same structures can arise *in vitro* and *ex vivo* by a detailed survey of assembly conditions.^35^ Importantly, all high-resolution cryo-EM structures of amyloid fibrils reported to date have described fibril structures at a single time point, either taken late in disease progression or post-mortem for *ex vivo* samples, whereas a single time point is used for fibrils assembled *in vitro*, purportedly representing the endpoint of assembly. That different fibril structures result by alteration of the solution conditions and factors such as agitation^26,35,36^ is an example of an assembly reaction under kinetic control (i.e., the most thermodynamically stable fibril is not necessarily the reaction product), by contrast with the behavior seen in most protein folding reactions.^37,38^ This raises the possibility that different fibril forms could develop during assembly, dependent on how quickly they nucleate/elongate relative to each other. Indeed, there is historical evidence that fibril properties can change during assembly, as demonstrated by atomic force microscopy of α-synuclein,^39^ nanobody labeling of α-synuclein^40^ and nuclear magnetic resonance of Aβ.^41^ Furthermore, Aβ fibrils at the center of plaques formed in mice models of Alzheimer’s disease show different dye-labeling behavior to those at their periphery, suggesting that fibrils can also mature over time *in vivo*.^42^ Importantly, because samples *ex vivo* rely on donors, the resulting amyloid structures represent the assemblies that are present in the final stages of disease progression, potentially overlooking transient and potentially biologically deleterious fibrillar assemblies. The progression of polymorphic structures and fibrillar intermediates is thus an important and poorly understood factor in understanding amyloid structures and their mechanism(s) of formation.

**Figure 1:**
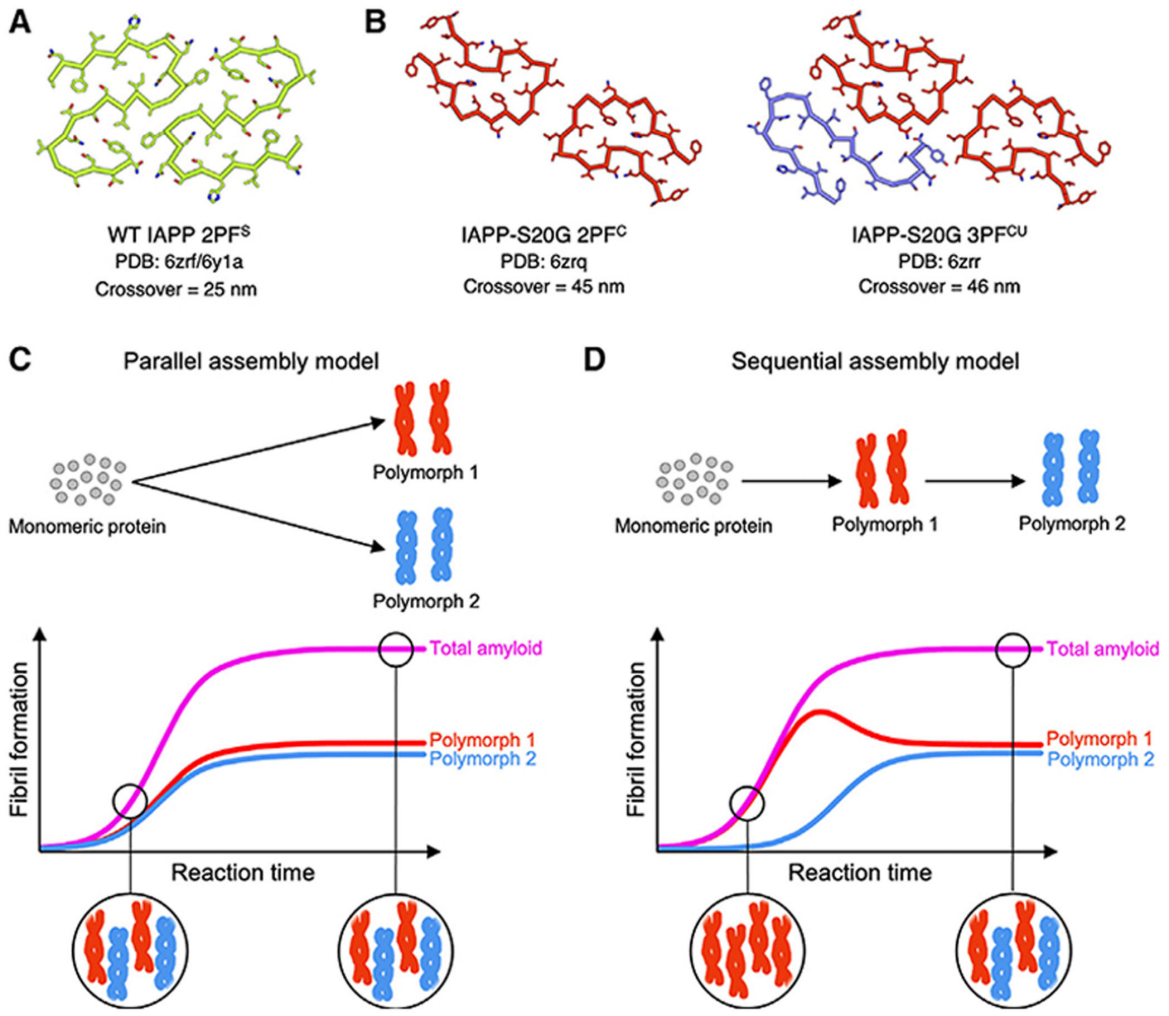
The assembly of polymorphic amyloid fibrils—does polymorphism change during the course of aggregation? (A–D) Cartoon views of the (A) WT hIAPP amyloid and (B) IAPP-S20G amyloid fibril structures formed *in vitro* published in our previous study,^46^ with nomenclature of the fibril type and chain coloring chosen to match those reported herein. Note that similar structures of WT IAPP fibrils have been reported by others.^29,48^ Simplified cartoon schemes for possible polymorphic fibril assembly processes via a (C) parallel or a (D) sequential model. Below each proposed model, illustrative cartoon plots are shown corresponding to different fibril assembly reactions that yield a similar mixed polymorph population at the end point. The typical sigmoidal growth for bulk amyloid formation (magenta, e.g., monitored by ThT fluorescence) can mask the underlying, potentially divergent assembly processes of individual polymorphs (red and blue). The schematics represent contrasting example cases from a spectrum of possible assembly mechanisms, and other models likely exist, including mixed parallel/sequential models and those including secondary nucleation on the fibril surface. This highlights the need to better understand the kinetic relationships between different structural polymorphs during an aggregation reaction. Determining whether polymorphism remains constant throughout self-assembly or different polymorphs appear sequentially is a crucial step toward that goal. See also Figure S8.

Human islet amyloid polypeptide (IAPP) is a 37-residue polypeptide hormone that is co-secreted with insulin from pancreatic β cells and plays an essential role in glycemic regulation. IAPP forms amyloid deposits in the pancreas of >90% of individuals with T2D.^43–45^ A single genetic mutation resulting in a serine to glycine substitution at residue 20 (IAPP-S20G) is associated with familial, early-onset T2D.^46,47^ Recently, cryo-EM structures of IAPP amyloid fibrils assembled *in vitro* for the wild-type (WT)^48–50^ sequence and IAPP-S20G^48^ have been determined, as well as those of patient-seeded WT IAPP.^30^ Interestingly, the IAPP-S20G variant generated two major fibril polymorphs, which are each structurally different to WT IAPP fibrils, with different subunit folds and fibril crossover lengths (~45 nm for IAPP-S20G and ~25 nm for WT)^48^ (Figures 1A and 1B). The IAPP-S20G fibril polymorphs share a conserved two-protofilament (2PF) core, with one polymorph containing an additional peptide chain to make a 3PF fibril in which the additional chain has a different fold to those in the 2PF core. Previous studies have shown that surface-mediated secondary nucleation dominates the *in vitro* fibrillation kinetics of both WT IAPP^51,52^ and IAPP-S20G.^52^ The interaction of monomers with the surface of preformed fibrils creates assembly-competent oligomers with a rate ~10^8^-fold more rapid than primary nucleation for both sequences.^52^ This led us to speculate that the IAPP-S20G 2PF form might appear early in assembly and catalyze the formation of the 3PF form. If so, a different ratio of 2PF:3PF fibrils should be observed at different times during the assembly reaction (Figures 1C and 1D). Negative stain EM (nsEM) showed that the first-formed fibrils of IAPP-S20G do not display an obvious twist, with visible crossovers appearing later during assembly.^48^ However, detailed analysis of the interplay of the time of fibril growth and the appearance of different fibril polymorphs could not be gleaned from these low-resolution observations.

Here, we have used cryo-EM to determine the structures of the fibrils present at three distinct time points during IAPP-S20G fibrillation, chosen to represent the different phases of fibril growth. These include an early time point in the lag phase of assembly, which samples the first fibrils formed, an intermediate time point during the exponential growth phase in which secondary nucleation and elongation dominate, and a late time point during the plateau phase, sampling the fibril products at an apparent steady state. Remarkably, different fibril polymorphs describe each phase of assembly, with some structures only present at certain times. In total, seven distinct fibril polymorphs were observed, including the two previously solved IAPP-S20G polymorphs (2PF and 3PF^48^), alongside five new structures that were not observed previously at a single reaction time point. These results demonstrate directly via high-resolution structures that fibril polymorphism changes during assembly and provide evidence of a potential structural progression, which shows that polymorphism is a complex, dynamic, and evolving landscape.

## Results

### IAPP-S20G fibril morphology evolves with time

**Figure 2:**
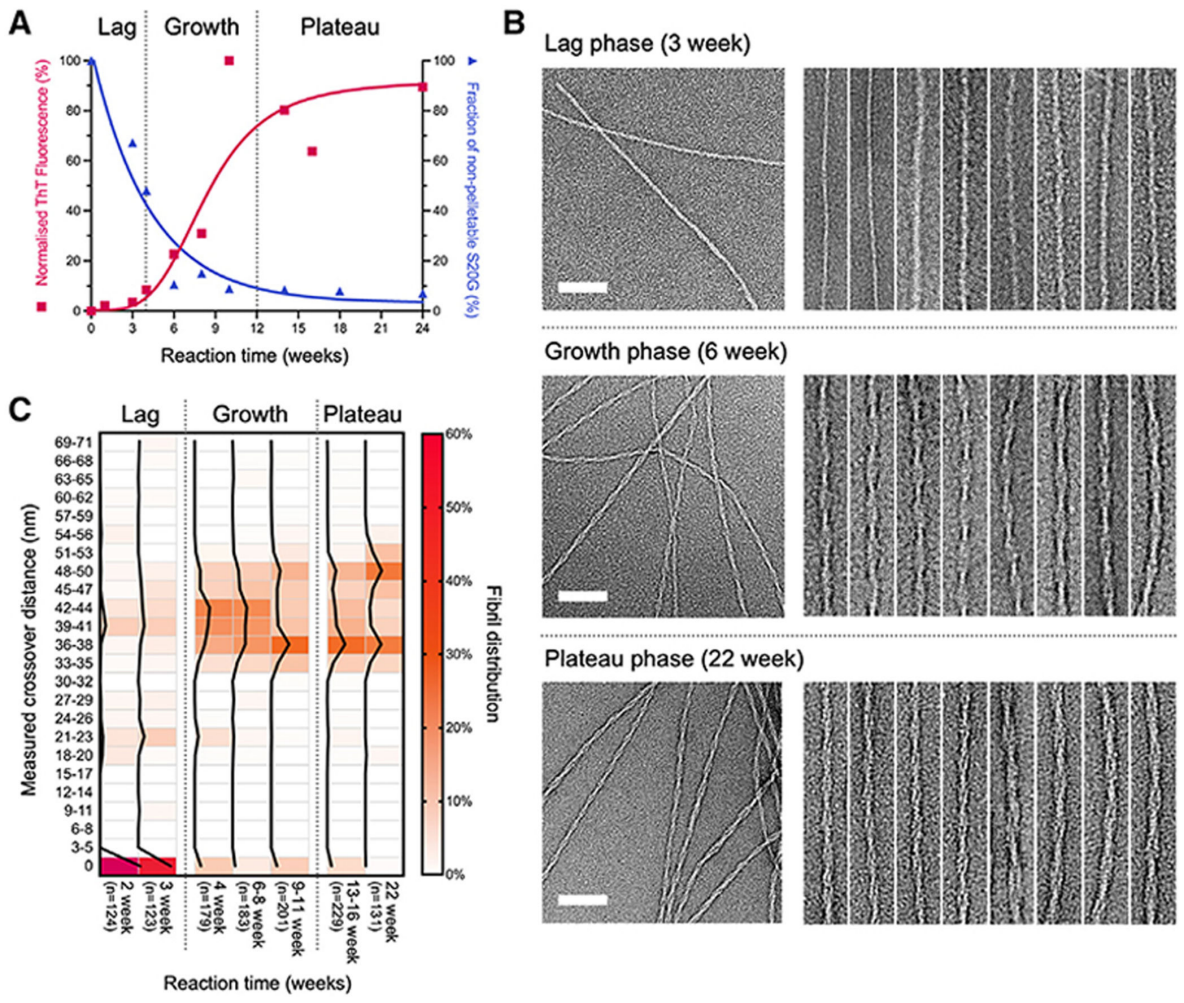
Initial characterization of IAPP-S20G fibril populations over time (A) Aliquots from fibrillation reactions were removed at different times and ThT fluorescence measured (red). hIAPP-S20G remaining in the supernatant after centrifugation (STAR Methods) was monitored in parallel using HPLC (blue). The time course highlights the lag, growth, and plateau phases of assembly and shows that early aggregated species either do not bind ThT or fluoresce only weakly in its presence. Peptide integrity was confirmed at the end of the reaction using LC-MS as shown in Figure S1. (B) Representative negative stain (ns)EM images of IAPP-S20G fibrils at different time points (3, 6, and 22 weeks) representing the lag, growth, and plateau phases of the ThT profile (scale bar represents 80 nm). Adjacent to each are zoomed sections from multiple nsEM images showing the diversity of fibril morphologies observed at each time point (box dimensions ~240 × 45 nm). Additional nsEM images from different time points are shown in Figure S2. (c) Heatmap of the percentage distribution of fibril crossovers measured from nsEM images of IAPP-S20G samples at different incubation times. Multiple fibrillation reactions contributed to each reaction time (except for the 22-week time point) as outlined in the STAR Methods (all reactions are individually plotted in Figure S3). The percentage of fibrils within each crossover length group is colored for each separate population, according to the displayed key, with a black line plot tracking these values for each time point. The number of fibrils measured for each time point is annotated on the x axis by “n.”

*In vitro* growth of IAPP-S20G fibrils was performed quiescently in glass vials, enabling samples to be extracted at different time points for analysis. Aliquots were removed at different times after initiating assembly and ThT fluorescence measured from a single vial to follow the course of assembly (Figure 2A, red curve). Under the conditions employed (STAR Methods), the t_1/2_ of amyloid fibril assembly is ~8 weeks, with distinct lag (0–4 weeks), growth (4–12 weeks), and plateau (12–24 weeks) phases. Mass spectrometry showed no degradation of the peptide during the time course (Figure S1). Estimates of fibril yield were also determined at each time point by pelleting aggregated material and quantifying the concentration of IAPP-S20G remaining in the supernatant using analytical high-performance liquid chromatography (HPLC) (STAR Methods) (Figure 2A, blue curve). Interestingly, this suggests that early fibril species do not label with ThT as effectively as those in the later stages of the assembly process, with no other types of aggregate visible in negative stain images (Figure S2A).

nsEM images of multiple reactions showed that oligomers or amorphous aggregates are not observed at any time point during aggregation (in these conditions), with the first fibrils visible between 2 and 3 weeks (note that oligomers of hIAPP >~50 kDa with an ordered structure should be observable by nsEM, using a Tecnai F20 microscope and Ceta detector) (Figure S2A). The majority of these early fibril species were untwisted (Figures 2B, 2C, and S2). Fibril appearance begins to change at around 4 weeks, after which time the majority of fibrils observed have distinct, measurable crossovers spanning ~33–50 nm (Figures 2C and S3). In our previous study,^48^ this was the point at which the two IAPP-S20G fibril polymorphs reported (Figures 1A and 1B) were solved. In samples measured between 9 and 22 weeks, a bimodal distribution of fibril types develops, with peak crossover distances around 36–38 and 48–50 nm, concurrent with a relative depletion of the fibrils with crossovers between these two lengths (Figures 2C and S3). These results were obtained from multiple reactions (with biological repeats for different time points, see STAR Methods), ensuring that the observed changes in morphology are reproducible and correlate with reaction progression. All four of the reactions imaged at 2–3 weeks showed that at least 40% (mean 55% ± 15%) of observed fibrils were untwisted and, in each reaction, this decreased to <7% (mean 5.2% ± 1.6%) in all later time points (Figures S2 and S3).

**Figure 3:**
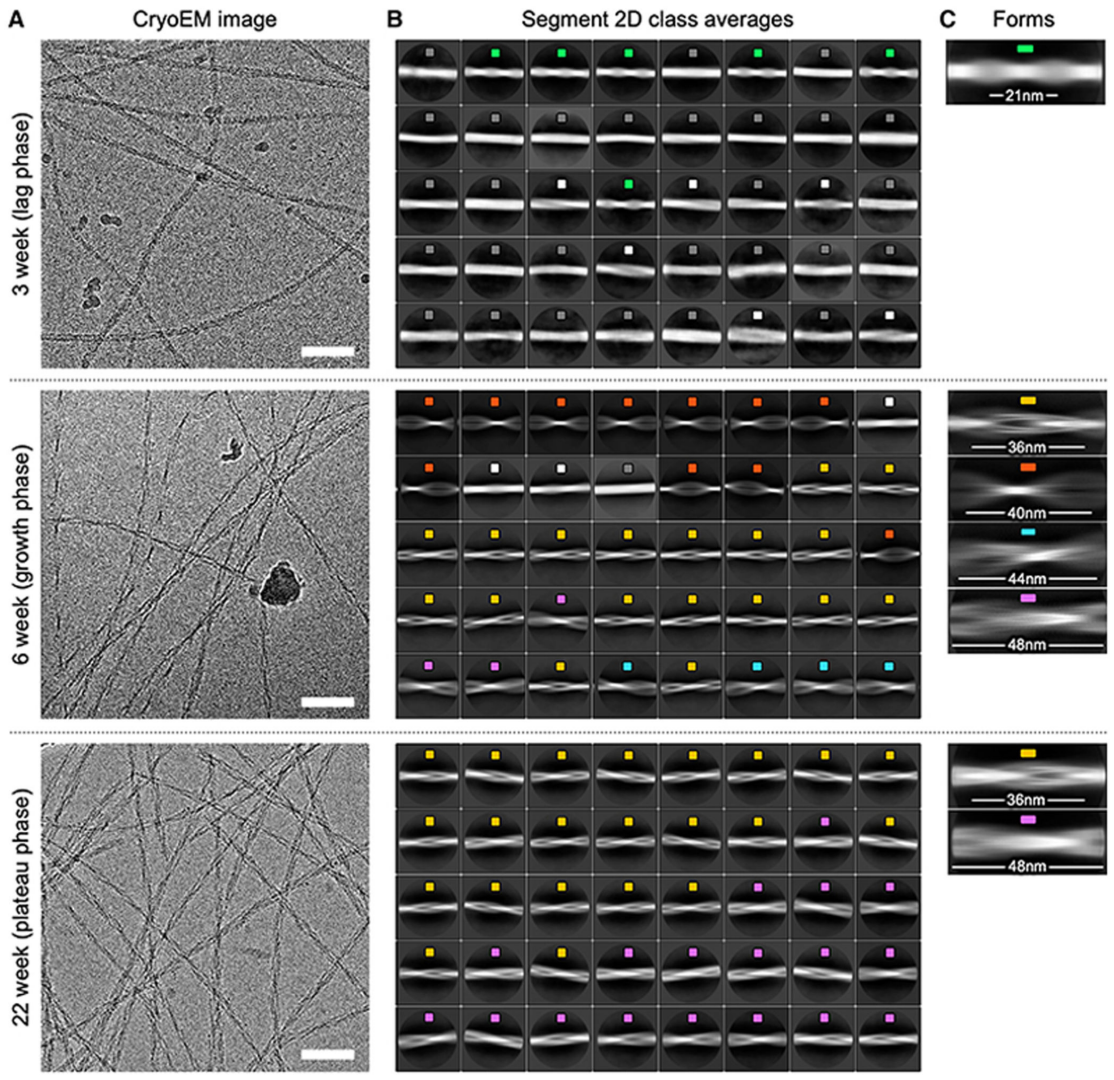
Different IAPP-S20G fibril populations are present in each phase of assembly (A) Representative cryo-EM images from each dataset collected for different IAPP-S20G fibrillation time points (3 week, lag phase; 6 week, growth phase; and 22 week, plateau phase). The scale bar (white) represents 100 nm. (B) The 40 most populated 2D class averages are shown for each dataset color coded by the apparent fibril polymorph determined by the crossover distance, ordered by class occupancy (top left to bottom right). Class averages with fibril segments showing no observable crossover features are labeled in dark gray, and those showing ambiguous internal features that could not be reliably grouped into other forms are labeled in white. Constructed using 23 binned particles of the full set of segments for each dataset after the removal of picking artifacts. The box size is ~50 nm. C) Zoomed images of selected 2D class averages from the datasets shown in (B), representing the different regular fibril forms initially identified. The measured fibril crossover distance is annotated for each. See Figure S4 for a comparison with 2D class averages from a replicate 3-week sample.

Based on these data, we selected three distinct time points to solve the fibril structures present to high resolution using cryo-EM: 3 weeks, representing the late lag phase, 6 weeks, representing the growth phase, and 22 weeks, representing the plateau phase (Figure 3A). Over 2,000 cryo-EM movies were collected across different areas of the grid to maximize the sampling of the fibril species present at each time point. 2D classification of fibril segments from the cryo-EM images revealed that ~30% of the fibrils in the 3-week dataset display an ~21-nm crossover distance, which was not a major component in the 6- or 22-week datasets (Figures 3B and 3C), consistent with the analysis of nsEM images (Figure 2C). The remaining fibril segments at the 3-week time point (~70%) were untwisted (labeled gray in Figure 3) or had no clear crossovers (labeled white in Figure 3). The fibril morphologies apparent were also different between the 6- and 22-week fibril populations (Figures 3B and 3C). The 6-week dataset was more polymorphic, with crossovers at distinct distances (36, 40, 44, and 48 nm), whereas only two of these were observed within the most populated 2D class averages obtained at 22 weeks.

### Fibrils at 3 weeks have a novel fold

**Figure 4:**
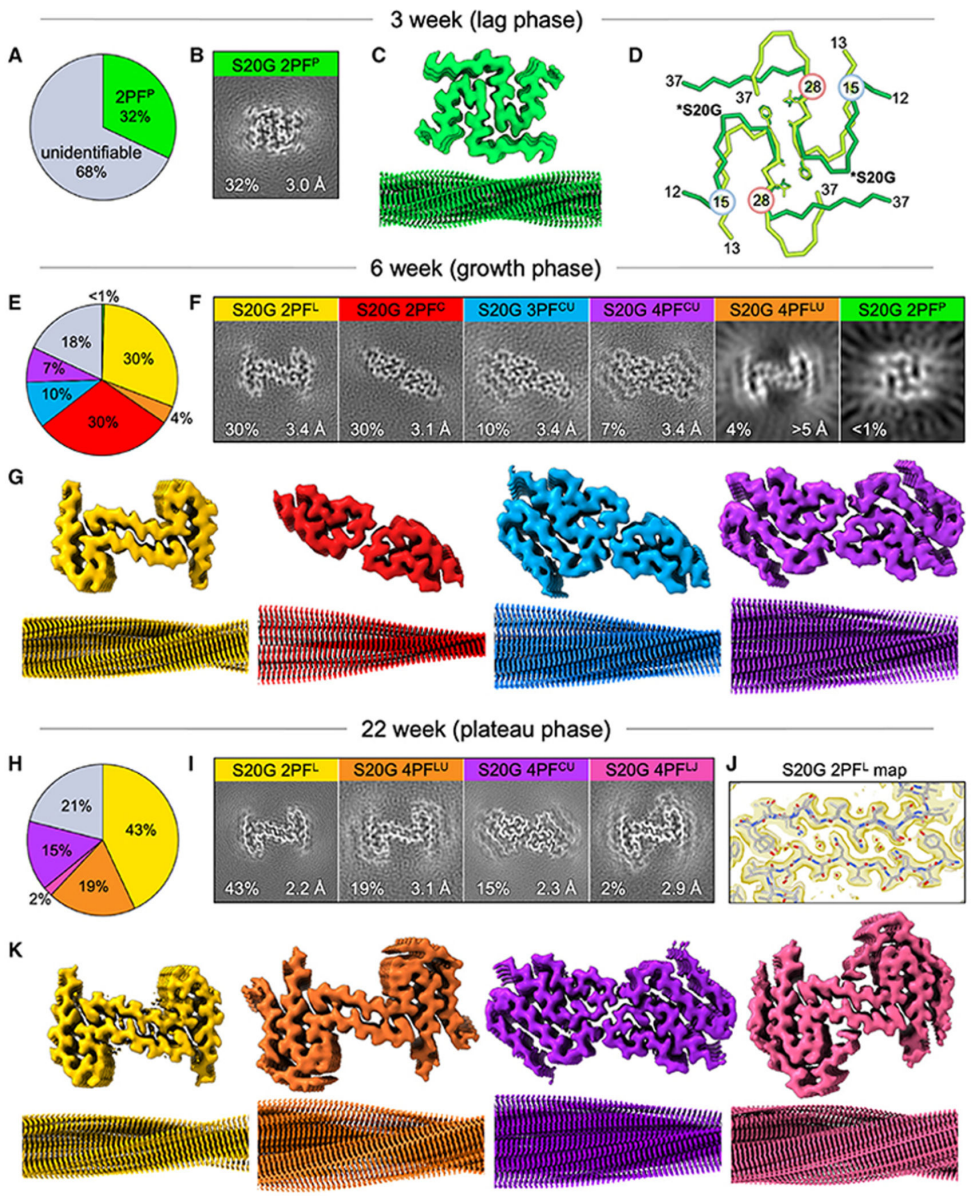
Cryo-EM structure determination reveals that multiple IAPP-20G fibril polymorphs differentially populate the different phases of assembly Pie chart of the fibril polymorph assignments from all the segments processed within the (A) 3-week, (E) 6-week, and (H) 22-week cryo-EM datasets of IAPP-S20G. The coloring matches the fibril polymorphs displayed throughout the figure and Figure 3, with gray representing unidentifiable fibril structures. Slice view of a layer of the final IAPP-S20G (B) 3-week, (F) 6-week, and (I) 22-week maps for each fibril polymorph, which were generated by averaging the central six slices, corresponding to an ~5 Å section of each map. Perpendicular views of the core and fibril surface of each of the deposited maps from the (C) 3-week, (G) 6-week, and (K) 22-week cryo-EM datasets. (D) Superposition of a single layer of the 3-week IAPP-S20G 2PF^P^ (PDB: 8awt, dark green) and the WT IAPP 2PF^S^ (PDB: 6zrf,^48^ light green) structures showing that they share a conserved inter-protofilament interface (involving residues ^23^FGAIL,^27^ shown as sticks) and subunit core fold between residues 15–28 (highlighted blue/red). The location of the mutation S20G is highlighted on the 2PF^P^ model. (J) Zoomed section of the 22-week IAPP-S20G 2PF^L^ map transparent to show modeling of an ordered water channel (red spheres) at the inter-protofilament interface. The colors used to denote each fibril type are consistent throughout all figures. See cryo-EM processing details in Figure S5 (3-week dataset), Figure S6 (6-week dataset), and Figure S9 (22-week dataset), respectively.

A single solvable fibril form representing ca. one third of all fibrillar IAPP-S20G imaged was present at 3 weeks (Figure 4A, see also Figures S5A–S5I). These fibrils yielded a 3.0 Å resolution cryo-EM map (Figures 4B, 4C, S5F, and S5G) that contains a 2PF fibril core with a 21 nm crossover distance (Figure 3C), into which an atomic model could be built for residues 12–37 of each subunit (Figure 4D). The core is arranged as two closely packed, symmetrical chains each with a P-shaped fold, which we term 2PF^P^. Interestingly, this polymorph is different to the previously published IAPP-S20G 2PF structure (PDB: 6zrq)^48^ (Figure 1B), which has a C-shaped core, and we hence-forth term 2PF^C^. It is also different to the S-shaped 2PF structure of WT IAPP fibrils (2PF^S^)^30,48,50^ (Figure 1A). Despite these differences, the 3-week IAPP-S20G and WT IAPP fibrils share some similarities: they have a similar crossover length (21 vs. 25 nm), have a conserved inter-subunit interface involving the major aggregation prone region of IAPP (residues 23–27), and share a similar backbone conformation across residues 15–28 (Figure 4D). However, the polypeptide termini pack differently in the IAPP-S20G 2PF^P^ and IAPP-WT subunit folds, mostly in the C-terminal region (residues 28–37), which is extended in IAPP-S20G but folds back to create the WT 2PF^S^ fold.

The remaining 68% of fibrils at the 3-week time point have no discernible twist, and their structure(s) could not be solved by helical reconstruction. Attempts to 3D classify these fibril segments using the IAPP-S20G 2PF^P^ structure, unfeatured cylinders or the multiple IAPP-S20G fibril structures solved at later time points (see below) as templates were unsuccessful (Figures S5H and S5I). The features of the 3-week fibril population were reproduced in cryo-EM data from a second IAPP-S20G reaction (Figures S4A–S4D). Most of the fibrils again have little or no twist. Although there were not enough particles to resolve the structure to high resolution in this dataset, similar 2D class averages and 3D classification maps at lower resolution (Figures S4B and S4C) suggest that 2PF^P^ was also present as an early fibril structure in this second reaction. This particular IAPP-S20G aggregation reaction was also sampled for the 22-week cryo-EM time point described later, validating a significant change in the fibril structures during assembly by comparing 2D class averages at 3 and 22 weeks from the same solution (Figure S4B).

### Six different IAPP-S20G polymorphs appear in the growth phase

The fibrils present after 6 weeks, which represents the growth phase of fibril formation (Figure 2A), are strikingly different to those observed at the 3-week time point (Figure 3). Nearly all (82%) of the fibrils have evident crossovers and can be assigned to a particular polymorph, with crossover spacings ranging from 36–50 nm. More surprisingly, and in marked contrast to the single structure solvable to high resolution at 3 weeks, six distinct fibril polymorphs could be identified at this time point (Figures 4E, 4F, and S6). The only ordered fibril form in the 3-week dataset, 2PF^P^, had almost completely disappeared, with <1% of the fibril segments at 6 weeks having the ~20 nm crossover distance consistent with 2PF^P^, and these were insufficient to generate a high-resolution 2PF^P^ structure (Figures S6A and S6B). However, four fibril structures could be solved to high resolution (3.1–3.4 Å) (Figures 4G and S6C–S6H). These showed the presence of two distinct structural families of fibril: those with an L-shaped subunit fold (2PF^L^ [30%]) and those with the C-shaped fold observed previously.^48^ These C-shaped fibrils included one with two protofilaments (2PF^C^ [30%]), one with three protofilaments (3PF^CU^ [10%]), and one with four protofilaments (4PC^CU^ [7%]). A sixth polymorph, identified as a second four protofilament structure, was too rare (~4%) to enable high-resolution structure determination in this dataset (Figure S6F) but was later resolved in the 22-week dataset (4PF^LU^; see below).

**Figure 5:**
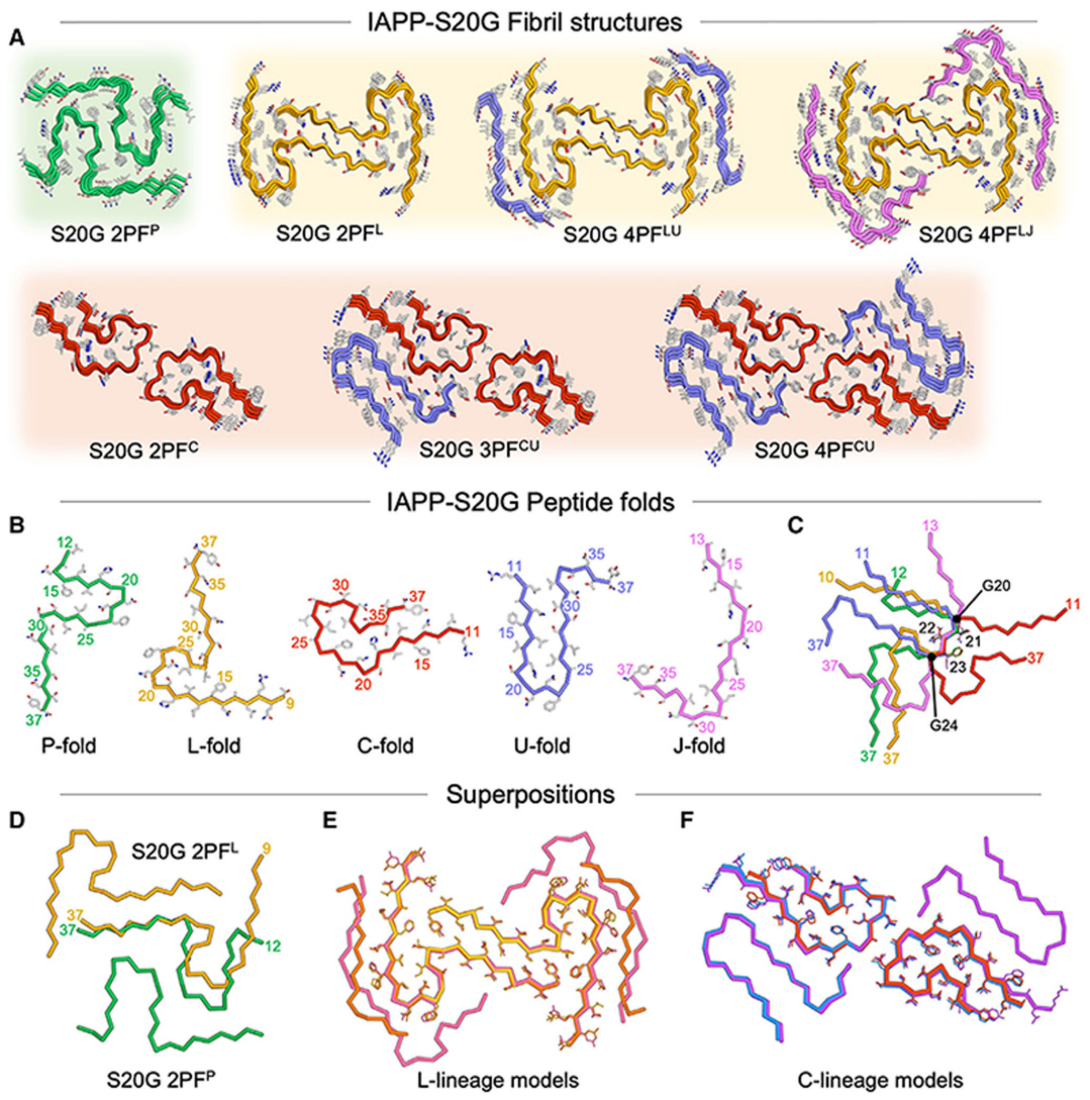
Different IAPP-S20G fibril structures and subunit folds observed during the fibrillation time course (A) Cartoon view of the seven unique IAPP-S20G fibril structures solved to high resolution. Peptide backbones are color coded in relation to the five different subunit folds present in the fibril assemblies (P, green; L, yellow; C, red; U, blue; and J, pink). The structures are grouped based on the fold of the 2PF core of the fibrils (P-lineage, green; L-lineage, yellow; and C-lineage, red). (B) Ribbon view of the five distinct IAPP-S20G subunit folds, colored as in (A). The N- and C-terminal residues ordered in each structure are numbered. (C) Superposition of the five different subunit folds, aligned on the structurally conserved sequence _20_GNNFG_24_ for which the side chains are displayed as sticks. (D) Superposition of 2PF^L^ (yellow) and 2PF^P^ (green), aligned on one of the peptide chains from each structure. The second chain of each fibril interacts on opposite sides of the superposed chain, highlight that the fibril architectures are very different.(G) (E) Superposition of one layer of each of the three L-lineage fibril structures, colored by structure (2PF^L^, yellow; 4PF^LU^, orange; and 4PF^LJ^, pink). The three structures share a conserved 2PF core (RMSD C_α_ atoms is 0.38 Å [54 atoms], 2PF^L^ vs. 4PF^LU^ and 0.44 Å [56 atoms], 2PF^L^ vs. 4PF^LJ^). (G) Superposition of one layer of each of the three C-lineage fibril structures, colored by structure (2PF^C^, red; 3PF^CU^, blue; and 4PF^CU^, purple). The three structures share a conserved 2PF core (RMSD C_α_ atoms is 0.39 Å [44 atoms], 2PF^C^ vs. 3PF^CU^ and 0.48 Å [40 atoms], 2PF^C^ vs. 4PF^CU^). See also Figure S7 to see the fit of each displayed model into its respective cryo-EM map and Figure S10 for further images of the L-lineage 2PF^L^, 4PF^LU^, and 4PF^LJ^ fibril assemblies. Both PyMol (Schrödinger) and ChimeraX^53^ were used for making structure figures.

Two of the polymorphs observed at 6 weeks are identical to those solved in our previous study,^48^ namely, 2PF^C^ and 3PF^CU^, and share a conserved 2PF core with C-shaped subunit folds (Figures 4G, 5A, 5B, and 1B). The higher-resolution maps obtained here revealed better resolved peptide backbone density than was possible in previous ~4 Å resolution maps^48^ (Figure S7 vs. S8), and correspondingly higher quality atomic models could be built. The newly observed polymorph 4PF^CU^ shares subunits in common with both 2PF^C^ and 3PF^CU^ but has a fourth, U-shaped protofilament, which stacks as a symmetry-equivalent subunit to the third protofilament of 3PF^CU^ (Figures 4G and 5A). These three forms constitute the C-lineage of IAPP-S20G polymorphs (Figure 5A), and, in the growth-phase sample, the 2PF^C^ polymorph is three times more prevalent than 3PF^CU^, which is, in turn, 1.5 times more prevalent than 4PF^CU^ (Figure 4F). With knowledge of this 4PF structure, re-analysis of our previously published dataset^48^ revealed the presence of a small population of 4PF^CU^, which was not resolvable previously (Figure S8). This provides independent validation of the robustness and reproducibility of the IAPP-S20G assembly process.

The remaining two novel IAPP-S20G polymorphs at 6 weeks constitute a distinct family of structures that we term the L lineage (Figure 5A). These include 2PF^L^ (30% populated) and a minor (4% populated), related, species 4PF^LU^ (Figure 4F). The L-shaped fold underlying the 2PF^L^ core in these two polymorphs has a different conformation to those of the C- and U-shaped subunits in the 2PF^C^, 3PF^CU^, and 4PF^CU^ structures (Figures 5A–5C). The L-shaped subunit fold most closely resembles the P-shaped 2PF^P^ fibrils observed in the lag phase, with subtle differences in the kinking of the two termini (Figure 5C). However, 2PF^P^ and 2PF^L^ fibrils are structurally distinct. The second protofilament stacks on a different side of the common core L/P subunit in each fibril, generating completely different inter-subunit interfaces (Figure 5D).

### Not all IAPP-S20G polymorphs persist in the plateau phase

Upon initial examination of the cryo-EM images for the 22-week sample, the fibrils appeared similar to those at 6 weeks, with almost all fibrils displaying ~36 or ~48 nm crossovers (Figure 3A). However, 2D classification revealed that the sample at 22 weeks is less polymorphic than at the 6-week time point (Figures 3B and 3C). Indeed, only three of the six polymorphs observed in the 6-week dataset prevailed in the 22-week dataset: 2PF^L^ (43%), 4PF^LU^ (19%), and 4PF^CU^ (15%), whereas there was no evidence for 2PF^P^, 2PF^C^, or 3PF^CU^ fibrils (Figures 4H, 4I, and S9). These datasets used an energy filtered detector, and the cryo-EM map resolutions were significantly improved to 2.2 Å for 2PF^L^ and 2.3 Å for 4PF^CU^ (Figures 4J, 4K, and S9), allowing for precise peptide modeling and direct visualization of the handedness of the fibrils (Figure S7). Ordered water molecules (Figures 4J and S9D) help to bridge the largely polar 2PF^L^ inter-protofilament interface, only the second such observation of an ordered solvent channel within a fibril core.^54^

Perhaps most strikingly, at the 22-week time point, the proportion of 2PF^L^ fibrils is increased (from 30% to 43%), accompanied by an increase in 4PF^LU^ fibrils (from 4% to 19%), such that these related fibril forms dominate (totaling 62%) the fibrils observed (Figures 4H and 4I). The increased proportion of 4PF^LU^ fibril segments enabled the structure of this fibril form to be solved to 3.1 Å resolution (Figures 4K and S9K). In this new structure, the 2PF^L^ core is decorated with two external chains with ordered residues 21–37, whose conformation resembles the U-subunit observed in the 3PF^CU^ and 4PF^CU^ polymorphs (Figure 5A). Low-resolution maps confirm the presence of the entire U-fold from residue 11 in the external chains of this fibril structure (Figures S10A and S10B). Remarkably, further structural variation also exists within the 4PF^L^ segments, with a minor subset (4PF^LJ^) containing an alternate peptide conformation for the two chains flanking the same conserved 2PF^L^ core (Figures 5A, S9H, and S10). The flanking J-shaped subunit fold spans residues 13–37 and is distinct from all other IAPP-S20G subunit folds described thus far (Figures 5B and 5C). This fourth, 22-week IAPP-S20G fibril structure represents just 2% of the total fibril segments, with a map solved to a resolution of 2.9 Å (Figures 4I, 4K, and S9I).

### Larger, more stable polymorphs appear as assembly progresses

**Figure 6:**
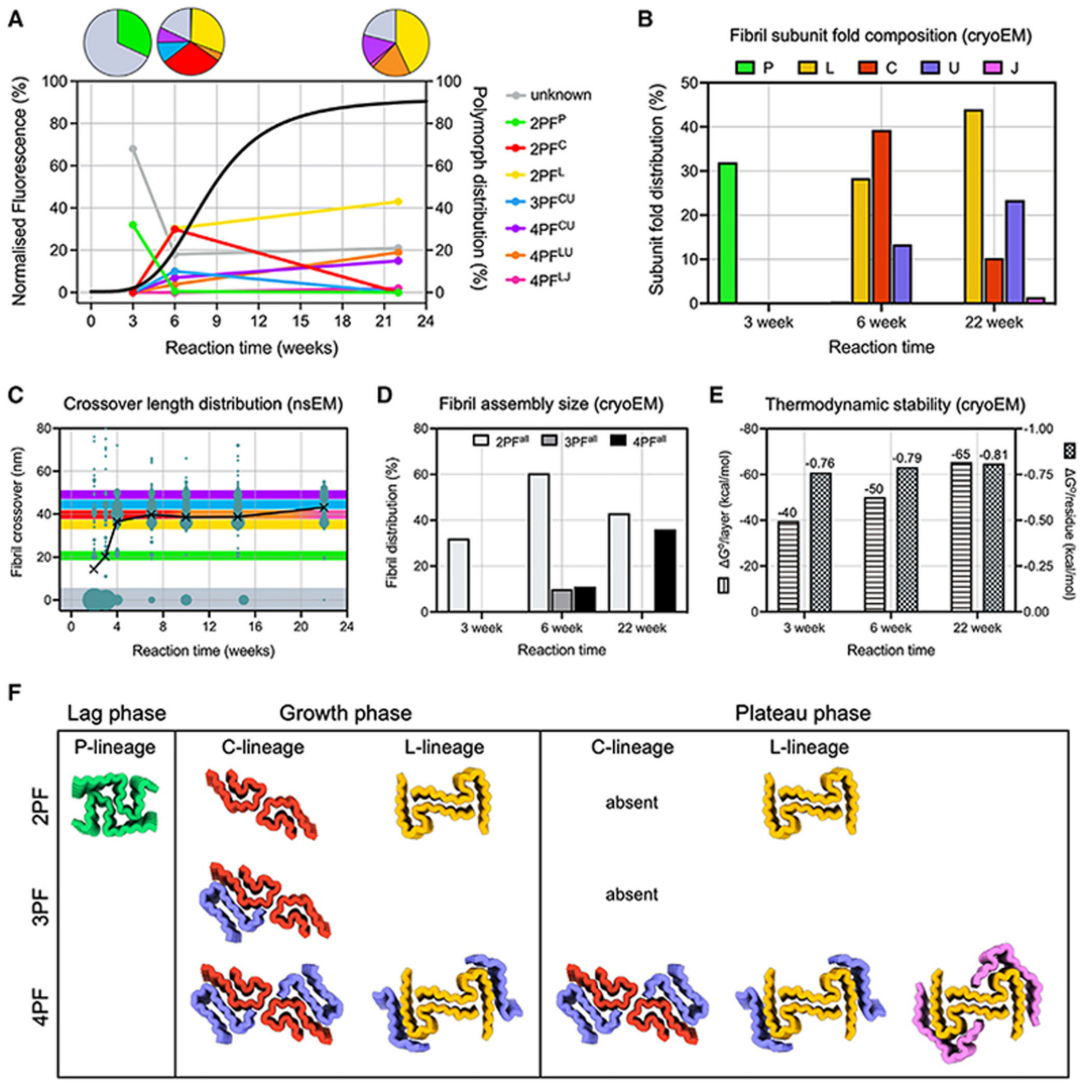
Summary plots describing the structural maturation of IAPP-S20G fibril polymorphs during the assembly time course (A) Line plot of the distribution of the different fibril polymorphs observed in each cryo-EM dataset (right y axis). For reference, the normalized ThT fluorescence from Figure 2A indicating reaction progression is plotted on the left y axis (black line). Pie charts above show the percent of each fibril type in the 3-, 6-, and 22-week samples. (B) Bar graph of the distribution of the different subunit folds observed within the fibrils from each cryo-EM dataset, calculated according to the distribution of polymorphs and number of each subunit fold per fibril layer. (C) Bubble plot of the fibril crossover distributions at different time points measured from nsEM images, using the same data as in Figure 2C. The size of the bubble relates directly to the number of fibrils with the same crossover and the average values for each time point are tracked with black crosses and a black line. The expected crossover range for the seven fibril polymorphs, colored as in (A) are shown as lines in each respective color. (D) Bar graph of the distribution of different sized fibril assemblies, including all 2PF, 3PF, and 4PF polymorphs for each cryo-EM dataset showing a shift from 2PF to 4PF fibril assemblies as fibril formation progresses. (E) Bar chart of the average ΔG°/layer (left y axis) and ΔG°/residue (right y axis) of fibrils at different time points based on the distribution of different polymorphs and the number of ordered residues in each layer of the fibril. The values for each polymorph were calculated as the average from two independent methods based on FoldX^55^ and described by Eisenberg/Sawaya^12,56^ (as shown in Figure S11 with further depiction of the calculated stability of each structure). (F) Summary scheme of the different fibril structures seen at each time point divided into the P-, C-, and L-lineages, respectively, showing a shift toward 4PF assemblies in the plateau sample.

A total of seven unique fibril structures were observed across the time points sampled here (Figure 5A), comprising different combinations of five unique subunit folds (Figure 5B). The structures can be divided into different lineages based on three different 2PF cores. 2PF^P^ does not form higher order assemblies, but 2PF^C^ resides within both 3PF and 4PF structures, whereas 2PF^L^ also resides within two different 4PF assemblies (Figure 5A). These 2PF cores superpose to the respective cores in the larger assembly states, with C_α_ RMSD values <0.5 Å in all cases (Figures 5E and 5F). The fit of models into sharpened cryo-EM maps, combined with these core superpositions, strongly suggests that all of the S20G structures described represent left-handed fibrils (Figures S7B and S7C, see STAR Methods). The distribution of fibril polymorphs changed dramatically during assembly (Figure 6A). Along with unresolved fibrils, 2PF^P^ appeared first, whereas few fibrils of this type persist in the growth phase, and none were observable in the plateau phase. By contrast, 2PF^C^ and 3PF^CU^ are observed in the growth phase but disappear in the plateau phase. Conversely, the 2PF^L^, 4PF^CU^, and 4PF^LU^ fibril polymorphs became more dominant in the plateau phase. The balance of sub-unit folds underlying the different fibril structures also changes as assembly progresses, with the P-fold found in the earliest fibrils in the lag phase, followed by the L- and C-subunit types in the 6-week sample, with the L- and U-subunit folds dominating at 22-week time point (Figure 6B). Larger fibril assemblies accumulate as assembly progresses, both in terms of the average repeating crossover length of fibrils (Figure 6C) and the average number of subunits per layer of the fibril, i.e., the number of proto-filaments in each fibril form (Figure 6D). For example, 36% of the dataset shows 4PF fibril structures in the 22-week plateau sample compared with 11% and 0% in the 6-week growth and 3-week lag phase samples, respectively.

To explore how the stability of the structures relates to the progression of assembly, the thermodynamic stability of each subunit fold and each fibril structure were calculated using two independent methods of free energy calculation (STAR Methods).^12,55,56^ The results showed that each subunit fold within each polymorph has a similar per residue free energy of ca. −0.8 kcal/mol (Figures 6E and S11), highlighting that an array of structures with similar stability are possible when an intrinsically disordered peptide, such as IAPP, assembles into a cross-β amyloid fold. Importantly, however, because there is a shift toward fibrils with a higher mass per unit length as assembly progresses (Figures 6D and 6F), the larger 4PF forms have a greater total calculated stability per layer of the fibril compared with the 3PF and 2PF forms (Figures 6E and S11). Therefore, for this amyloid assembly reaction, both kinetic and thermodynamic factors appear to influence polymorphism as the system moves toward equilibrium, generating larger fibril assemblies from subunits that are structurally conserved and individually isoenergetic.

### Fibril maturation is a generic property of hIAPP aggregation

To probe whether fibril structure changes during assembly reactions of a different IAPP sequence, we set up an assembly time course of WT hIAPP in similar buffer conditions but in a 96-well plate with continuous measurement of the fluorescence of neighboring ThT reporter wells (Figure S12A). Samples (from reactions without ThT) were taken at different time points in duplicate and analyzed using cryo-EM (STAR Methods). Again, a shift in fibril architecture was observed, from a mixture of fibrils with unresolvable structure(s) at early times, to a more homogeneous population dominated by a single defined fibril architecture in the plateau phase of assembly (Figures S12B–S12D). Fibrils with a 25 nm crossover were observed, similar to that of the previously solved 2PF^S^ form of WT hIAPP fibrils,^30,48,50^ but the final solution of this fibril has a similar backbone conformation to that seen in a polymorph generated by seeding hIAPP with *ex vivo* seeds from the pancreas of a donor with type II diabetes (the TW2 polymorph^30^). The results highlight the importance of solution conditions and vessel type in determining the fibril structures formed (plate vs. Eppendorf or glass vials, as was used in previous studies^30,48,50^). Notably, the new WT hIAPP fibril polymorph identified here at steady state was present in the earlier time point analyzed but was drastically increased in proportion as the reaction progressed (14% ± 3% to 45% ± 3%). These results therefore again show that the extent and nature of IAPP fibril polymorphism changes over time and suggest that such change is a conserved feature of amyloid formation of IAPP and possibly other proteins.

## Discussion

High-resolution cryo-EM structures of amyloid to date have typically focused on fibril morphology at a single time point. *In vitro*, this is typically a time at which fibrils have grown and display a repeating twist, enabling their structure(s) to be solved using helical processing.^57^ For studies of amyloid *ex vivo*, an end point in disease is typically set by the availability of donor tissue. Here, we have built on our previous work on IAPP-S20G amyloid formation^48^ and used cryo-EM to describe the different fibril structures within a heterogeneous, dynamic ensemble of polymorphs at different stages of an amyloid assembly reaction. The results are striking because, although it has been demonstrated previously for both Aβ and α-synuclein that the structural properties of fibrils can change over time,^39–42^ we reveal the details of how the 3D structures of hIAPP fibrils progressively evolve during amyloid formation.

In the lag phase, as IAPP-S20G fibril assembly begins, only one polymorph with a regular helical twist is observed, namely, 2PF^P^. This two-protofilament fibril structure shares some features with the 2PF^S^ fibrils observed previously for WT hIAPP^48,50^ but is structurally distinct (Figure 4D). Although fibril structures of WT and S20G IAPP have yet to be solved *ex vivo*, four structures have been observed by seeding monomeric WT hIAPP *in vitro* with material extracted from the Islets of Langerhans of a donor with T2D.^30^ Interestingly, the subunit fold of 2PF^P^ resembles the subunits within the *ex vivo* seeded WT hIAPP amyloid with polymorphs TW1 and TW3, described by Cao et al.^30^ (Figure S13). Seeded *ex vivo* WT hIAPP (TW4) also contains elements of structure reminiscent of those in the L-, U-, and C-folds of IAPP-S20G fibrils described here (Figure S13). Importantly, 2PF^P^ is not the only fibril formed in the lag time because the majority (68%) of fibrils lacked a regular helical twist, such that their structures could not be solved. Nonetheless, the fact that these fibrils lack an obvious twist demonstrates that they are structurally distinct from all of the twisted polymorphs solved herein. It is noteworthy that such fibrils appear when ThT fluorescence is low relative to the amount of pelletable material, suggesting that these fibril forms may not bind ThT or bind in a manner in which ThT fluoresces relatively weakly, consistent with these assembles having a different structure to those resolved at later time points. Different intensities of ThT fluorescence have been reported previously for different fibril morphologies.^58–61^ We cannot conclude how these early structures evolve as assembly progresses, i.e., how later fibril species form. Nonetheless, the results clearly demonstrate that fibril structure changes with time.

The new polymorphs that appear in the growth phase fall into two distinct two-protofilament forms, 2PF^C^ and 2PF^L^, with different subunit structures and intermolecular interactions. However, they share the ability to accrete new subunits to form additional protofilaments that lead to the generation of higher order fibril assemblies (Figure 6F). This principle was suggested in our earlier work that first revealed the structures of 2PF and 3PF S20G-IAPP fibrils,^48^ but that study lacked the temporal (and spatial) resolution of the present study. Here, we describe structures that show that subunit accretion is a generic property of *in vitro* IAPP-S20G fibril assembly. These structures highlight the remarkably plasticity of the IAPP-S20G sequence, whereby the same minimal 25 residues (amino acids 13–37) can adopt at least five different subunit conformations. Accretion of different subunits then builds larger fibril assemblies by different packing of these five subunit folds.

In the plateau phase, subunit accretion appears to be a slow but stabilizing force that shifts amyloid assembly toward thicker fibrils (i.e., fibrils with more protofilaments) over time. This is accompanied by a subtle lengthening of the crossover distance, presumably as a slight unwinding of the fibril structure more easily accommodates the extra mass per unit length of thicker fibrils.^25,62^ This is most dramatically demonstrated by the complete disappearance of 2PF^C^ and 3PF^CU^ fibrils in the 22-week sample, which represented ~40% of the total dataset at 6 weeks, with 4PF^CU^ being the only C-lineage fibril remaining by 22 weeks. The L-lineage fibrils undergo a similar trend, with two distinct 4PF^L^ forms found in the plateau phase (4PF^LU^ and 4PF^LJ^). However, by contrast with the C-shaped fibrils, 2PF^L^ remains highly populated at this late stage in assembly as the single remaining 2PF form.

**Figure 7:**
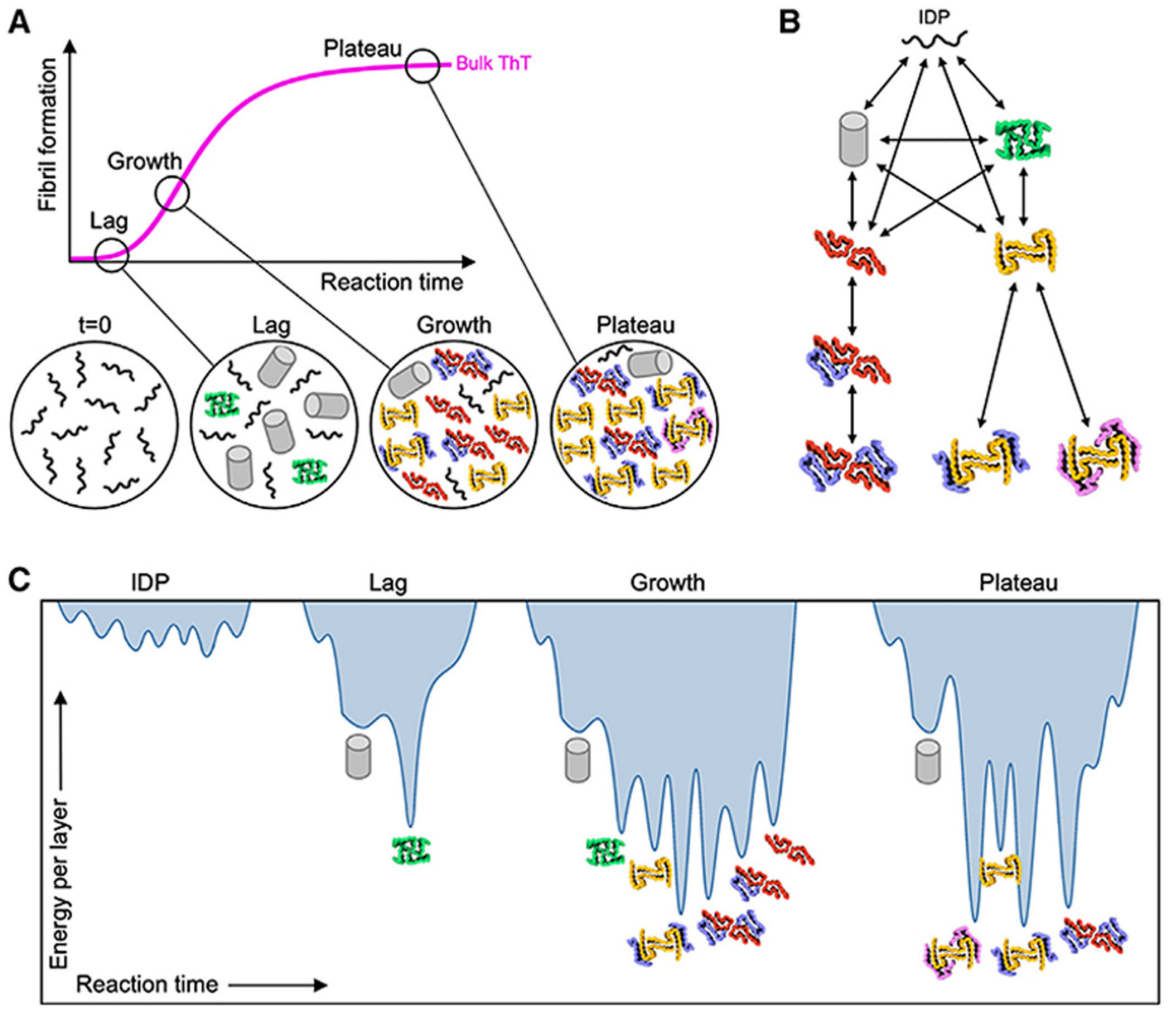
Cartoon summary of IAPP-S20G polymorph progression with a proposed mechanism of different kinetic landscapes during the different stages of assembly (A) Cartoon ThT plot of the IAPP-S20G time course in the style of Figures 1C and 1D with windows representing the different ratios of species observed at each stage of assembly. Black squiggles represent unstructured monomer, gray tubes represent untwisted fibrils, and the resolved fibril polymorphs are represented by their respective core structures, colored by subunit fold as in Figure 6. (B) Potential assembly pathway schematic based on the fibril architectures and order of appearance. Each step is presumed to be reversible, and all plausible transitions have been included. It should be noted that other intermediates may exist that have not been captured, the untwisted fibrils may contain a mixture of states, and that monomer could directly contribute to all of the fibril assemblies. (C) Illustrative energy landscapes for each stage of IAPP-S20G assembly, based on the observed structures and their calculated stability based on a single fibril layer to scale each peak height. Each peak is labeled by the fibril structure it represents. Not intended as a comprehensive depiction but rather a potential snapshot of the kinetically accessible states at each assembly stage. See also Figure S11.

The thermodynamic properties of a polypeptide fold are a significant driving force in the formation of amyloid fibrils.^12,63,64^ However, fibril polymorphism is generally recognized as being under kinetic, rather than thermodynamic, control, with the ratio of products being determined by the rate at which they form and not necessarily by their thermodynamic stability.^37^ Free energy calculations^12,55^ show that all fibril structures solved here have similar per residue energy scores. Hence, although thermodynamic stability dictates which structures are possible, i.e., it creates the wells in the energy landscape for aggregation, the relative population of each polymorph is not dictated by this stability but instead by the rate at which a particular fibril polymorph forms. Why then does polymorphism change with time during amyloid growth (Figure 7A)? The rate of fibril formation is dependent on multiple processes, including primary nucleation, secondary nucleation, fragmentation, and elongation, each with a different dependency on monomer concentration. The contribution of each process to fibril growth will therefore change during assembly as the populations of monomers and fibrils change. In the lag phase, high monomer concentrations and low fibril concentrations favor primary nucleation, whereas secondary nucleation dominates during the growth phase (with elongation important throughout assembly). On this basis, we propose that the first fibril form observed, 2PF^P^, is kinetically the most accessible when primary nucleation dominates. Thereafter, fibril formation is driven predominantly by secondary processes, which for IAPP-S20G can occur 10^8^-fold more rapidly than primary nucleation events.^52^ As such, in the growth phase, we propose that 2PF^P^, which is the only IAPP-S20G 2PF species in this study that does not generate higher order 3PF/4PF assemblies, then becomes overwhelmed by, and might catalyze, the formation of new polymorphs via secondary nucleation (Figure 7B). The observation that the calculated average ΔG° per molecule increases as 3PF and 4PF assemblies form (Figures 6E and S11) suggests that a drive toward increasing thermodynamic stability may promote formation of these larger assemblies when the reaction reaches steady state. Whether further changes in structural polymorphism arise at even longer times remains an open question, but should such changes happen, they will likely occur over extended timescales that were not accessible here. The data suggest, therefore, that each stage of IAPP-S20G assembly reflects different kinetic landscapes that lead to different polymorphs being favored at different times (Figure 7C).

The results presented reveal two fascinating features of amyloid assembly that have not been fully recognized hitherto. First, we provide a molecular mechanism by which larger fibril assemblies could be formed (Figure 7B), by accretion of subunits that dock against the preformed surfaces of their predecessors. The low abundance of 3PF and 4PF species in patient-derived samples of Tau, Aβ, and α-synuclein observed to date (all of which assemble from an initially intrinsically disordered polypeptide) suggests that this feature could be disfavored *in vivo*, possibly by the binding of molecular chaperones or other macromolecules to the fibril surface (reviewed in Ulamec et al.^65^). Determination of the structures of WT IAPP or IAPP-S20G fibrils *in situ* (using electron tomography) or *ex vivo* (using cryo-EM) will help answer this question. Second, we show that time is a crucial, but under-studied, factor in amyloid formation and hence, possibly, also in the progression of amyloid disease. For IAPP, polymorphism changing over time appears to be a general property of amyloid assembly, with both WT hIAPP IAPP-S20G fibrils shown to mature from largely unresolvable fibril types into defined structured polymorphs. Most notably, we demonstrate directly using cryo-EM structure determination, that the early stages of IAPP-S20G amyloid assembly reproducibly contain a fibril polymorph (2PF^P^) that is structurally distinct from those at later times. A recent report capturing cryo-EM fibril structures during *in vitro* tau fibril assembly that finishes with a disease-relevant polymorph recapitulates these key findings in another amyloid system, whereby many structured fibril intermediates form and disappear as assembly progresses.^61^ This raises the possibility that the early stages of amyloid assembly *in vivo* may contain currently unmapped fibril architectures with potentially different pathological properties to those observed to date.

## Limitations of the study

In this work, we show that hIAPP amyloid polymorphism changes during the assembly process, presenting a high-resolution study of fibril maturation, which has revealed the unexpected finding that fibril structures formed early in assembly are structurally different to those seen late in the assembly process. Experimentally, obtaining consistent behavior working with amyloid formation is challenging, and therefore, there is a limitation to the level of quantitative analysis that can be drawn comparing percentages of a polymorph at one time versus another. A major question arising from our study is whether different polymorphs directly interconvert, as would be formally possible for the maturation of 2PF^C^ fibrils into 4PF^CU^ fibrils in the plateau phase, or whether fibril interconversion requires disassembly into monomer or transient oligomeric species prior to their reassembly into a new amyloid fold. Future studies that enable individual fibrillar species to be tracked in real time, for example by developing fibril-specific probes, will be needed to answer this question. Additionally, further work exploring the seeding potential of fibrils formed at the different stages of assembly, and their stability against depolymerization, denaturation, and fragmentation, would build on the structural insights presented here and provide a more detailed mechanistic understanding of assembly. Investigating structures formed during the assembly of other amyloidogenic proteins will help to determine whether changing polymorphism over time is a fundamental concept in amyloid formation *in vitro*, whereas similar experiments performed in cells or animal models will be required to determine whether and how fibrils mature in a biological setting. Finally, how fibrils of different structure affect cellular homeostasis and potentially the onset of dysfunction and disease are additional important outstanding questions. Such work will be critical for future therapeutic studies of diseases associated with amyloidosis. In that light, we note that the structures of WT IAPP and IAPP-S20G from human tissue remain to be determined, and the structures of the *in vitro* assembly reactions described here may not necessarily be the same.

## Star⋆Methods

## Key Resources Table

**Table:** 

REAGENT or RESOURCE	SOURCE	IDENTIFIER
Chemicals, peptides, and recombinant proteins		
IAPP-S20G peptide sequence (C-terminal amidation)	Synthesised	KCNTATCATQRLANFLVHSSNNFGAIL SSTNVGSNTY
WT IAPP peptide sequence (C-terminal amidation)	Synthesised	KCNTATCATQRLANFLVHSGNNFGAIL SSTNVGSNTY
Hexafluoroisopropanol (HFIP)	Sigma Aldrich	105228-110G
ThioflavinT (ThT)	Sigma Aldrich	T3516-25G
Ammonium acetate	Sigma Aldrich	A2706-100ML
PAL-NovaSyn TG resin	Merck	8551370005
Fmoc-Ala-Thr(psiMe,Mepro)-OH	Merck	8521800005
Fmoc-Ser(tBu)-Ser(psiMe,Mepro)-OH	Merck	8521870005
Fmoc-Leu-Ser(psiMe,Mepro)-OH	Merck	8521790005
Deposited data		
IAPP-S20G 3 week cryoEM dataset (FT24)	EMPIAR	EMPIAR: 11714
IAPP-S20G 3 week cryoEM dataset (FT14)	EMPIAR	EMPIAR: 11716
IAPP-S20G 6 week cryoEM dataset (FT11)	EMPIAR	EMPIAR: 11715
IAPP-S20G 22 week cryoEM dataset (FT14)	EMPIAR	EMD: 11717
IAPP-S20G 3 week 2PF^P^ fibril model	PDB	PDB: 8AWT
IAPP-S20G 3 week 2PF^P^ fibril cryoEM map	EMDB	EMD: 15696
IAPP-S20G 6 week 2PF^C^ fibril model	PDB	PDB: 8AZ1
IAPP-S20G 6 week 2PF^C^ fibril cryoEM map	EMDB	EMD: 15729
IAPP-S20G 6 week 2PF^L^ fibril model	PDB	PDB: 8AZ0
IAPP-S20G 6 week 2PF^L^ fibril cryoEM map	EMDB	EMD: 15728
IAPP-S20G 6 week 3PF^CU^ fibril model	PDB	PDB: 8AZ2
IAPP-S20G 6 week 3PF^CU^ fibril cryoEM map	EMDB	EMD: 15730
IAPP-S20G 6 week 4PF^CU^ fibril model	PDB	PDB: 8AZ3
IAPP-S20G 6 week 4PF^CU^ fibril cryoEM map	EMDB	EMD: 15731
IAPP-S20G 22 week 2PF^L^ fibril model	PDB	PDB: 8AZ4
IAPP-S20G 22 week2PF^L^ fibril cryoEM map	EMDB	EMD: 15753
IAPP-S20G 22 week 4PF^CU^ fibril model	PDB	PDB: 8AZ5
IAPP-S20G 22 week 4PF^CU^ fibril cryoEM map	EMDB	EMD: 15754
IAPP-S20G 22 week 4PF^LU^ fibril model	PDB	PDB: 8AZ6
IAPP-S20G 22 week 4PF^LU^ fibril cryoEM map	EMDB	EMD: 15755
IAPP-S20G 22 week 4PF^LJ^ fibril model	PDB	PDB: 8AZ7
IAPP-S20G 22 week 4PF^LJ^ fibril cryoEM map	EMDB	EMD: 15756
Negative stain images (all IAPP-S20G reactions)	University of Leeds Data Repository	https://doi.org/10.5518/1230
ThT fluorescence, fibril yield and all plotted data	University of Leeds Data Repository	https://doi.org/10.5518/1230
Software and algorithms		
EPU V3.0	ThermoFisher	https://www.thermofisher.com/uk/en/home/electron-microscopy/products/software-em-3d-vis/epu-software.html
RELION 4.0	Kimanius et al.^66^	https://relion.readthedocs.io/en/release-4.0/Installation.html#download-relion
CTFFIND 4.16	Rohou and Grigorieff^67^	https://grigoriefflab.umassmed.edu/ctf_estimation_ctffind_ctftilt
crYOLO V1.7	Wagner et al.^68^	https://cryolo.readthedocs.io/en/stable/installation.html
COOT V0.89	Emsley et al.^69^	https://www2.mrc-lmb.cam.ac.uk/personal/pemsley/coot/binaries/release/
PHENIX V1.17.1	Adams et al.^70^	https://phenix-online.org/download/
Molprobity	Williams et al.^71^	http://molprobity.manchester.ac.uk/
Fiji	Schindelin et al.^72^	https://imagej.nih.gov/ij/
Prism9	GraphPad	https://www.graphpad.com/how-to-buy/
ChimeraX-1.5	UCSF^56^	https://www.cgl.ucsf.edu/chimerax/download.html
PyMol V2.3.2	Schrödinger	https://pymol.org/2/
Anaconda (Python3)	ANACONDA	https://www.anaconda.com/download
FoldX	FoldX consortium (EMBL)	https://foldxsuite.crg.eu/
Eisenberg/Sawaya free energy calculation	Bash script provided by Michael Sawaya, UCLA^12^	https://people.mbi.ucla.edu/sawaya/amyloidatlas/
MassLynx V4.1	Waters UK	https://www.waters.com/waters/en_US/MassLynx-MS-Software/nav.htm?locale=en_US&cid=513662
Other		
Kinetex EVO C18 column	Phenomenex	N/A
Titan Krios G2 electron microscope	ThermoFisher	N/A
Falcon4 detector	ThermoFisher	N/A
Falcon4 with selectris energy filter	ThermoFisher	N/A
F20 electron microscope	Tecnai	N/A
Ceta CMOS detector	FEI	N/A
Clariostar platereader	BMG Labtech	N/A
Xevo QToF G2-XS mass spectrometer	Waters UK	N/A
Liberty Blue automated microwave peptide synthesizer	CEM Microwave Technology	N/A
Nexera LC-40 HPLC instrument	Shimadzu	N/A
Sephacryl S-100 HR resin	Cytiva	N/A

## Resource Availability

## Lead contact

Further information and requests for resources and reagents should be directed to and will be fulfilled by the lead contact, Prof. Neil Ranson (n.a.ranson@leeds.ac.uk).

## Materials availability

This study did not generate new unique reagents.

## Data and code availability

CryoEM data have been deposited at the EMPIAR, EMDB and PDB respectively and are publicly available as of the date of publication. Accession numbers are listed in the key resources table. Source data files, including ThT data, fibril yield data and negative stain images, are deposited and freely available at the University of Leeds Data Repository (DOI https://doi.org/10.5518/1230).

This paper does not report original code.

Any additional information required to reanalyse the data reported in this paper is available from the lead contact upon request.

## Experimental Model and Study Participant Details

The peptide used in this study was chemically synthesised, no cell lines or animal models were used.

## Method Details

### Synthesis of IAPP-S20G

IAPP-S20G with an amidated C-terminus was synthesised using a Liberty Blue automated microwave peptide synthesizer (CEM Microwave Technology) on a 0.1-mmol scale, as described previously.^46^ Briefly, 9-fluorenylmethyloxycarbonyl (Fmoc)-protected amino acids and PAL-NovaSyn TG resin (Novabiochem®, Merck) were used. Two pseudoproline dipeptides (Fmoc-Ala-Thr(psiMe,MePro)-OH and Fmoc-Leu-Ser(psiMe,Mepro)-OH, Merck) were used for the synthesis of Ala-8 and Thr-9, and Leu-27 and Ser28. All the residues and the two pseudoproline dipeptides were double coupled. Upon completion, the peptide was cleaved from the resin in a cleavage cocktail of trifluoroacetic acid (TFA, 9.4 ml), 3,6-dioxa-1,8-octanedithiol (250 μL), H2O (250 μL) and trii-sopropylsilane (100 μL). The mixture was stirred at room temperature for 3.5 h, concentrated under a nitrogen stream and the crude peptide precipitated in cold diethyl ether, followed by three washes with the same solvent. After dissolving in a 50% (v/v) acetonitrile aqueous solution containing 0.1% (v/v) TFA, the peptide was lyophilized. Subsequently, the crude was dissolved in 50% (v/v) DMSO aqueous solution to promote intramolecular Cys2-Cys7 disulfide bond formation. The oxidised peptide was purified by reverse-phase high-performance liquid chromatography (HPLC) (Kinetex™ EVO C18 column, Phenomenex) using a gradient of acetonitrile with 0.1% (v/v) formic acid. The mass of the purified peptide (3873.3 Da) was confirmed by ESI-MS and purity assessed to be >95% by analytical HPLC. After purification, peptide was again lyophilised and stored at -20°C until use.

### Synthesis of WT IAPP

For the plate-grown WT IAPP reactions (used for Figure S12), the same synthesis procedure was used as outlined for IAPP-S20G, except that Ser-20 was used in place of Gly-20, and Ser-19 and Ser-20 were coupled as an additional pseudoproline dipeptide (Fmoc-Ser(tBu)-Ser(psiMe,Mepro)-OH, Merck). Subsequent work-up, oxidation, purification, and quality control were the same as for IAPP-S20G, except for the different molecular weight (3903.3 Da) used in mass-directed HPLC and ESI-MS. After quality control, peptide was split into 200 μg aliquots in 0.1% formic acid, lyophilised, and stored at -20°C until use.

### IAPP-S20G fibrillation reactions

Lyophilised IAPP-S20G peptide was monomerized by dissolution in hexafluoroisopropanol (HFIP, Sigma-Aldrich) at a concentration of 256 μM and incubation for 30 min at room temperature. The solution was then aliquoted into 1.5 mL glass vials and the solvent completely evaporated to leave a peptide film around the walls of the container by blowing a gentle stream of nitrogen gas whilst gently swirling the vial. Each vial contained 59 μg of peptide as calculated by resuspending one vial and determining the concentration of peptide. This was done by measuring the absorbance at 280 nm in a spectrophotometer and using the calculated IAPP-S20G extinction coefficient of 1615 M^-1^cm^-1^. Frozen IAPP-S20G peptide films were allowed to thaw at room temperature for 5 min before gentle resuspension in 450 μL of assembly buffer (20 mM ammonium acetate, pH 6.8, filtered immediately prior to use) to a monomeric IAPP-S20G concentration of 30 μM. The suspension was filtered using a SpinX nitrocellulose-membrane filter (Corning) with centrifugation at 10,000g for 5 min and the eluate transferred to a clean glass vial. The samples were incubated quiescently at room temperature for the duration of the reaction time course. Aliquots were removed from multiple reactions at different time points for various experiments with respective reactions used as long as the remaining solution volume exceeded 200 μL. Where referenced in the methods and supplementary information, the identity of which IAPP-S20G reaction was used for which experiment is stated using the identifier FTXX (fibril tube, followed by a number).

### ThT experiments

For sampling the ThT fluorescence of the IAPP-S20G time course (Figure 2A), all readings were conducted over two plate-reader runs, each with different time points and a negative control without fibril sample was used to check for consistent fluorescence intensities between runs. At the time of each run, aliquots were removed from multiple glass vial reactions aged for different lengths of time to record the ThT fluorescence, detailed as follows: Run1, 0 week (FT31), 3 week (FT30), 6 week (FT29), 8 week (FT28), 10 week (FT27), 24 week (FT22); Run2, 1 week (FT33), 4 week (FT32), 14 week (FT29), 16 week (FT28). For each reading, 50 μL aliquots of the respective reaction were mixed with 10 μM ThT in 20 mM ammonium acetate pH 6.8 (75 μL total volume, 20 μM IAPP-S20G monomer equivalent) and fluorescence was measured in 96-well half-size plates (Costar) with the fluorescence emission at 460-510 nm (10 nm slit width) recorded after excitation at 440 nm (10 nm slit width) at a temperature of 25°C in a ClarioStar plate-reader (BMG Labtech). A ThT control without fibril added was used to scale the fluorescent signal (for measurements conducted on different days) and all measurements were blank-subtracted and normalised against the maximum recorded fluorescence.

For the continuous WT IAPP ThT fluorescence assay (Figure S12A), four aliquots of lyophilised peptide were collectively resolubilised in 0.5 mL 0.1% formic acid, loaded onto a Tricorn 10/300 GL Sephacryl S-100 HR column (Cytiva) equilibrated in 1 mM HCl (pH 3.0), and purified by size exclusion chromatography. Fractions containing monomeric IAPP were collected and quantified by UV absorbance at 280 nm using the calculated extinction coefficient of 1615 M^-1^.cm^-1^. IAPP eluate was combined with water and stocks of ammonium acetate, ThT, NaOH, and NaCl to yield 30 μM WT IAPP in 20 mM ammonium acetate with 1 mM NaCl and 10 μM ThT. The NaOH and NaCl stocks were included to neutralise the 1 mM HCl used in purification, and make the resulting NaCl concentration up to a controlled value, respectively. The correct final pH (6.8) was confirmed empirically. Reactions were set up in a clear-bottomed half-area 96-well plate with a low-binding surface (Corning 3881), 100 μL per well. The fluorescence data were collected and corrected as described above, but with triplicate reactions measured continuously every 10 mins. Each replicate was individually normalized relative to its own maximum and minimum fluorescence values. Fibrils for cryoEM analysis (Figures S12B–F) were grown in parallel, identical reactions on the same plate as the ThT assay but without ThT added.

### Fibril yield by HPLC

The following reactions were used for sampling different time points: 0 week (FT31), 3 week (FT30), 4 week (FT27), 6 week (FT29), 8 week (FT28), 10 week (FT27), 14 week (FT26), 18 week (FT27), 24 week (FT22), with largely the same reactions and times sampled as used for the ThT fibril assembly curve (Figure 2A). For each, a 60 μL aliquot of the respective fibril reaction was removed at the specified time and centrifuged at 17,000 g for 10 min to pellet aggregated material. After centrifugation, each supernatant was extracted and stored at -20°C until required. All the collected supernatant samples were subjected directly for analysis using HPLC (Nexera LC-40, Shimadzu). The sample was injected into a Nucleosil 300 C4 column (5 μm, dimensions 250 × 4.6 mm) and eluted with a gradient made by acetonitrile with 0.1 % (v/v) TFA and H_2_O with 0.1% (v/v) TFA. Triplicate calibration runs with known concentrations of the peptide between 0.5-32 μM were used to generate a calibration curve (available at Leeds Data repository, DOI https://doi.org/10.5518/1230) to calculate the concentrations of IAPP-S20G in each sample based on the observed peak fluorescence emission intensity at 306 nm (with excitation at 276 nm).

### Peptide integrity by mass spectrometry

A 60 μL aliquot of a fibril reaction at 22 weeks was collected and centrifuged at 17,000 *g* for 10 min. The supernatant was removed and the pellet resuspended in 1 mL HFIP to depolymerise aggregates. The HFIP mixture was incubated at 4°C for 24 h and then dried over a gentle stream of nitrogen gas to form a film of peptide around the wall of an Eppendorf tube. The film was then resuspended in 30 μL of 50% (v/v) acetonitrile aqueous solution with 1% (v/v) formic acid. As a control, a 30 μL aliquot of fresh IAPP-S20G peptide was similarly treated but using the entire sample without the centrifugation step. The samples were subsequently analysed by ESI-MS recorded using a Xevo QToF G2-XS mass spectrometer (Waters UK, Manchester, UK) operated in positive ion mode. Data were processed using MassLynx V4.1 supplied with the mass spectrometer.

### Negative stain EM

For the preparation of grids for imaging by negative stain EM, 4 μL of the respective IAPP-S20G fibrillation reactions (as identified in Figures S2 and S3) were applied to 300 mesh continuous carbon grids for ~1 minute. The samples were blotted, washed with water and then stained with 1% (w/v) uranyl acetate. Grids were imaged using a Tecnai F20 microscope with FEI Ceta CMOS detector at a magnification 50,000x (2 Å/pixel). For each sample, multiple images were collected at around -3 to -5 μm defocus.

### IAPP-S20G cryoEM sample preparation and data collection

The respective IAPP-S20G fibrillations were applied to Tergeo plasma cleaned (Pie Scientific) Lacey carbon 300 mesh grids (Agar Scientific). Undiluted sample was used for the 3 week time point, 1 in 2 diluted for the 6 week and 1 in 6 diluted sample for the 22 week time point. Dilutions were made using the fibrillation buffer. Each grid was blotted and frozen in liquid ethane using a Vitrobot Mark IV (FEI) with a 5 s wait and 5 s blot time respectively. The Vitrobot chamber was maintained at close to 100% humidity and 8°C. The cryoEM datasets were collected at the University of Leeds Astbury Centre using a Titan Krios electron microscope (Thermo Fisher) operated at 300 kV with a Falcon4 detector in counting mode. For the 6 week (IAPP-S20G, reaction FT11) dataset, a nominal magnification of 96,000x was set yielding a pixel size of 0.84 Å. A total of 2,350 movies were collected with a nominal defocus range of -1.3 to -2.5 μm and a total dose of ~43 e^-^/Å^2^ over an exposure of 5 s, corresponded to a dose rate of ~6.4 e^-^/pixel/s. For the 3 week (IAPP-S20G, reaction FT24) and 22 week (IAPP-S20G, reaction FT14) datasets, a nominal magnification of 130,000x was set yielding a pixel size of 0.9 Å and a Selectris energy filter (Thermo Fisher) was used operating with a 10 e^-^V slit. A total of 3,318 (3 week) and 4,373 (22 week) movies respectively were each collected with a nominal defocus range of -1.0 to -2.4 μm and a total dose of ~39 e^-^/Å^2^ over an exposure of 5 s, corresponded to a dose rate of ~6.3 e^-^/pixel/s. The repeated 3 week (IAPP-S20G, reaction FT14) dataset was collected at a magnification of 130,000x with the upgraded Falcon4i software yielding a pixel size of 0.95 Å and the Selectris energy filter was operated with a 10 e^-^V slit width. A total of 2,317 movies were collected, the nominal defocus range was -1.4 to -2.6 μm, total dose was ~43 e^-^/Å,^2^ exposure was 5 s and dose rate ~7.7 e^-^/pixel/s.

### WT IAPP cryoEM sample preparation and data collection

For each of the two replicates at each of the two time points studied from the 96-well plate-based WT IAPP fibril growth, an entire 100 μL reaction without ThT was removed. The fibrils were immediately pelleted (16,000 g, 10 mins), 90 μL of the supernatant (containing unaggregated peptide) removed, the pellet resuspended after addition of 90 μL of the reaction buffer (20 mM ammonium acetate, 1 mM NaCl) and the centrifugation repeated. The final pellet was resuspended in a total of 25 μL (early time points) and 50 μL (late time points) of reaction buffer and flash-frozen in liquid nitrogen until required. Thawed, undiluted sample was applied to Lacey carbon grids as described in the preceding section.

The cryoEM datasets were collected at the University of Leeds Astbury Centre using a Titan Krios electron microscope (Thermo Fisher) operated at 300 kV with a Falcon4 detector in counting mode. For the Repeat 1 cryoEM datasets at 5 and 23 h, a nominal magnification of 96,000x was set yielding a pixel size of 0.83 Å. A total of 4,995 (5 h) and 2,919 (23 h) movies were collected with a nominal defocus range of -1.4 to -2.6 μm and a total dose of ~39 e^-^/Å^2^ over an exposure of 4 s, corresponded to a dose rate of ~6.8 e^-^/pixel/s. For Repeat 2 cryoEM datasets at 5 and 23 h, a nominal magnification of 130,000x was set yielding a pixel size of 0.945 Å and a Selectris energy filter was used operating with a 10 e^-^V slit. A total of 2,973 (5 h) and 2,592 (23 h) movies were collected with a nominal defocus range of -1.3 to -2.5 μm and a total dose of ~40 e^-^/Å^2^ over an exposure of 5 s, corresponded to a dose rate of ~7.1 e^-^/pixel/s.

### IAPP-S20G CryoEM data processing

The following general processing scheme was applied to each of the four IAPP-S20G datasets with care taken to try to search for, classify and identify every distinct helical fibril type observed within each respective dataset. The raw EER movies were fractionated, aligned and summed using motion correction in RELION-4^66^ with a dose per frame of 1.0, 1.1, 1.4 and 1.0 e^-^/Å^2^ for the 3 week (FT24), 3 week (FT14), 6 week (FT11) and 22 week (FT14) datasets respectively. CTF parameters were estimated for each micrograph using CTFFIND4.^67^ Fibrils from roughly 100 micrographs were manually picked, and the fibril segments were extracted and used to train a separate picking model for each dataset in crYOLO^68^ for the automatic picking of all the micrographs. An exception to this was the two 3 week datasets, which contained a lot of empty images without fibrils and so were picked manually to facilitate removal of the empty micrographs. For extraction, an inter-box spacing of 19 Å(4× asymmetric units) was used and the resulting segments were initially extracted 2x binned using 300 pixel^2^ box sizes, with 160,648, 198,029, 842,792 and 1,978,281 segments for the 3 week (FT24, Figure S5), 3 week (FT14), 6 week (FT11, Figure S6) and 22 week (FT14, Figure S9) IAPP-S20G datasets respectively. At least two rounds of 2D classification were performed to remove picking artefacts such as carbon edges and ice contamination, keeping all classes that could be protein fibrils. At this stage, all of the selected 141,716 (3 week, FT24) and 1,489,798 (22 week) segments were extracted unbinned with a 300 pixel^2^ box size. For the highly polymorphic 6 week dataset, 2xbinned 2D class averages were used to split the data into two with 21,003 ~20 nm crossover potential 2PF^P^ segments separated from the remaining 697,471 segments with 36-50 nm crossover distances. Both subsets were then extracted unbinned with a 300 pixel^2^ box size.

In this study, likely due to the similar crossover distances and conserved structural elements in different IAPP-S20G fibril polymorphs, 3D classification was more effective at separating the different polymorphs completely, often with multiple classifications required due to the distinct helical parameter requirements of each polymorph. For the first round of 3D classification on each unbinned segment dataset, initial models obtained from 2x binned 2D class averages using the relion_helix_inimodel2d^57^ command were used as starting templates with helical twists derived from the corresponding measured crossover distances. An orientation sampling interval of 1.8° was used in combination with a strict high-resolution limit of 5 Å, t-value of 30 and a fixed helical rise of 4.8 Å to facilitate faster polymorph assignment and separation. For later classification rounds, with homogeneous segment sets, the orientation sampling was increased to 0.9°, t-value lowered to 20 and high-resolution limit increased to 4 Å. Throughout, separate test 3D classification runs with searches of both the helical rise and twist were run on subsets of the segments to feed improved helical parameters and maps back into the full classification/refinement scheme. Once a good model with resolved helical layers could be obtained from such 3D classifications, the homogeneous segments were put through 3D refinement, typically with finer helical searches of the twist and rise, template low-pass filter of 6 Å, initial sampling interval of 0.9° and a t-value of 10-15. CTF refinement and Bayesian polishing were then employed, before a final 3D refinement to give the following maps with resolutions based on the gold-standard 0.143 FSC cutoff: 3 week IAPP-S20G 2PF^P^ at 3.0 Å (Figures S5F and S5G); 6 week IAPP-S20G 2PF^L^ at 3.4 Å, 2PF^C^ at 3.1 Å, 3PF^CU^ at 3.4 Å and 4PF^CU^ at 3.4 Å (Figures S6G and S6H); 22 week IAPP-S20G 2PF^L^ at 2.2 Å (Figure S9D), 4PF^CU^ at 2.3 Å (Figure S9G), 4PF^LJ^ at 2.9 Å(Figure S9I), and 4PF^LU^ at 3.1 Å (Figures S9K and S9L). Further collection and processing details can be found in Table S1 (3 week datasets), Table S2 (6 week dataset) and Table S3 (22 week dataset).

### WT IAPP CryoEM data processing

The four WT IAPP cryoEM datasets were similarly processed to classify the entire fibril segments population observed in the data. The raw EER movies were fractionated, aligned and summed using motion correction in RELION-4 with a dose per frame of 1.0-1.1 e^-^/Å^2^. Empty and carbon-dominated images were rapidly removed by combining a RELION Select job (paneling micrographs at a display scale of 0.2x) with low-pass filtered micrographs generated by crYOLO. Similar to above, crYOLO was trained to pick the fibrils in the micrographs, with 192,721 (5 h, repeat 1), 143,882 (5 h, repeat 2), 444,036 (23 h, repeat 1) and 174,481 (23 h, repeat 2) segments extracted 2x binned with 300 pixel^2^ rescaled box sizes. The 2D classification runs were done using the 2x binned segments, removing only non-fibrillar artefacts. with the most populated classes from one repeat of each of the final round of classification displayed in Figure S12C. Despite multiple attempts with different templates generated from single 2D class averages (relion_helix_inimodel2d) and with unfeatured cylinders, only one of the polymorphs could be resolved by 3D classification, so the segment distribution pie and bar chart (Figures S12B and S12D) values are based on assignment from the morphologies apparent in 2D class averages. The discovered polymorph, was further processed following similar steps to those described in the previous section. The best map obtained at 4.1 Å is displayed (Figures S12E and S12F) with a refined helical twist of 178.23°and rise of 2.41 Å respectively, however the density for the stacked β-strand layers along the fibril axis could not be completely resolved (with areas of fused density between layers, Figure S12F), meaning that a definitive solution was not obtained. Many different variations of helical symmetry, including trying rises of ~4.8 and ~9.6 Å and using the previously solved WT IAPP 2PF^S^ structure (PDB: 6ZRF)^46^ and associated helical parameters as a starting template failed to yield a better solution.

### IAPP-S20G model building

For each of the nine deposited cryoEM maps, a corresponding protein model was constructed or docked, iteratively edited in Coot^69^ and real-space refined in Phenix^70^ before repeating the model to cover three fibril layers and performing a final real-space refinement in Phenix with NCS restraints. Where possible, starting templates for peptide chains were employed using the published IAPP-S20G 2PF structure^48^ (PDB: 6zrq) as well as the structures solved within this study. Otherwise, chains were built de novo in coot. The fit of the final built models to their respective EM maps is showcased in Figure S7, with the final model statistics assessed using MolProbity^71^ detailed in Table S1 (3 week dataset), Table S2 (6 week dataset) and Table S3 (22 week dataset).

### Fibril hand inference from cryoEM maps and prior studies

The hand of all polymorphs observed in the IAPP-S20G time course was shown to be left-handed by a variety of observations. Our previous study used AFM to observe that the 2PF^C^ and 3PF^CU^ polymorphs are left-handed. The high resolution cryoEM maps for 2PF^L^ and 4PF^CU^ (2.2 and 2.3 Å respectively) in this study resolved the orientation of backbone carbonyls in the peptide bonds to validate modelling these fibrils as left-handed (Figure S7B). With similar fibril appearances and crossover distances, it is hard to definitively tell 4PF^LU^ and 4PF^LJ^ fibrils a part from eachother as well as from the dominant 2PF^L^ fibrils observed in the late time point, complicating hand-determination by AFM and cryoET approaches. The resolutions of their maps are also not as high (3.1 and 2.9 Å, respectively), although a combination of sharpened cryoEM maps (Figure S7B) and the structural superposition of the 2PF core of the structures with 2PF^L^ (Figure 5E) offer strong supporting evidence that 4PF^LU^ and 4PF^LJ^ fibrils are also left-handed. Finally, the handedness of the early S20G polymorph 2PF^P^ is similarly difficult to resolve due to its short, subtle crossover and 3.0 Å resolution cryoEM map. Again, the use of a further sharpened map suggests a left-handed fibril backbone fits the density well, however to be more certain we also flipped the hand of the final cryoEM map and built a right-handed fibril model to inspect and compare both possibilities (Figure S7C). Several different regions of the map are compared and validation statistics calculated using Molprobity after building the right-handed model in Coot and passing it through 5 cycles of real-space refinement in Phenix. Both of which, signify that the 2PF^P^ fibril polymorph is almost certainly left-handed.

### Calculation of cryoEM fibril polymorph distributions

For each dataset in this study, the quoted polymorph distributions were determined using segment numbers from all 3D classification maps that showed fibril core slices that clearly corresponded to the same polymorph, at the point where further classification produced only classes displaying that polymorph. This was calculated as a percentage from all of the starting fibril segments that came out of the final cleaning 2D classification step. For example, for the 3 week dataset the first 3D classification showed 50% of segments relating to the 2PF^P^ polymorph (Figure S5B), these segments were then selected and 3D classified a second time whereby 44,654 segments (59% from this run) classified as 2PF^P^ (Figure S5C). The resulting selected 2PF^P^ class was 3D classified a third time (Figure S5E) and this time all the output classes displayed a 2PF^P^ core. As such, all of the 44,654 segments that went into that third classification were assigned to 2PF^P^ out of the total unbinned segment pool of 141,716, giving a distribution of 32% for that dataset. Fibril type assignment for the quoted distributions in the study was regardless of the resolution of the output classes and solely relied on the apparent fibril core morphology in the 3D class averages. On the other hand, for high-resolution structure determination, each polymorph segment pool was then divided further so that only the most ordered segments went into the final refined structures (22,801 segments out of 44,654 for 2PF^P^ in the example above).

### Stability calculations

For FoldX^55^ thermostability calculations, the deposited 5-layer fibril models for each structure were first optimised using the FoldX optimize command and then ΔG° values obtained with the stability command for the middle fibril layer. For Eisenberg thermostability calculations, shell scripts using the same calculations as in Sawaya et al.^12^ (used for the website Amyloid Atlas) were kindly provided by Michael Sawaya (University of California, Los Angeles) and the same 5-layer fibril models were used to generate ΔG° values for the middle fibril layer. In both cases, average stability per residue values were generated by dividing the layer free energy value of each polymorph by the number of residues ordered in the respective model. For 4PF^LU^, low resolution 3D classes showed that a longer stretch (residues 11-37) of the U-fold subunit was clearly present than built in the final structure (residues 22-37) (Figure S10). So, to avoid underinflating the stabilities of the 4PF^LU^ structure, the calculations were done using the docked 11-37 residue stretch of the U-fold subunits, taken from the high resolution 4PF^CU^ model. As the two methods calculate slightly different values for each polymorph, the average of the two stability values is used for each polymorph when referenced in the text and Figures 6E, 7C, and S11.

### Quantification and Statistical Analysis

Where reported (error bars in Figure S12B) ± error values and error bars represent standard deviations from all the values in the respective data group. For the measurement of fibril crossover lengths (used in Figures 2C, 6C, S2C, and S3), distance measurements were carried out manually in Fiji^72^ from negative stain images using the calibrated pixel size. Each measurement contributing to the “n=” values reported for each sample represents a single crossover value from a single fibril, whereby n is the number of fibrils measured. The single crossover value represents an average distance from measuring at least three consecutive repeats along the fibril and is estimated to the nearest nm. Prism 9 (GraphPad software, USA) was used for all data plotting in this work.

## Supplementary Material

Supplementary Figures

## Highlights

- IAPP-S20G amyloid fibril polymorphism drastically changes as assembly progresses
- Different fibril structures populate the lag, growth, and stationary phases of assembly
- Early populations contain transient species that disappear by the end of the reaction
- Structural lineages are proposed from common fibril cores in increasing assembly sizes

## In brief

Cryo-EM sampling of a diabetes-associated variant of islet amyloid polypeptide during the lag, growth, and stationary phases of fibril assembly reveals the dynamic nature of amyloid polymorphism over time.
