# Supplementary material for: Structural evolution of fibril polymorphs during amyloid assembly
Source: Cell. Author manuscript; Available in PMC 2025 May 22. (PMC7617692; doi:10.1016/j.cell.2023.11.025)
Supplement: Supplementary Figures [file EMS205469-supplement-Supplementary_Figures.pdf]

Supplemental figures

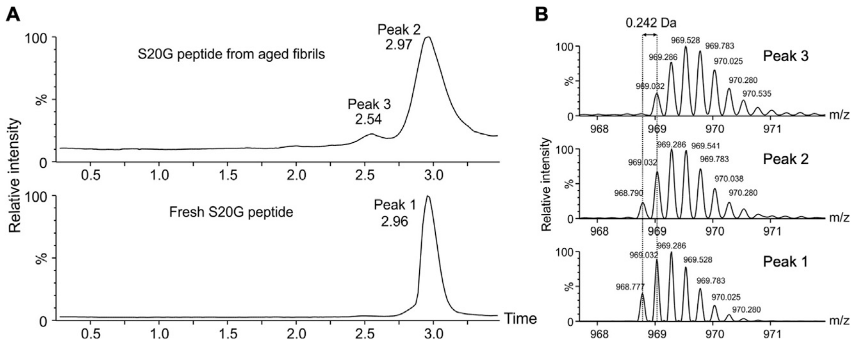

Figure S1. Analysis of peptide integrity during fibril growth assays, related to Figure 2 and STAR Methods

(A) HPLC trace of fresh (bottom trace) and 22-week aged (upper trace) IAPP-S20G and their retention times.

(B) Mass comparison of the IAPP-S20G monomer^{4+} from peaks 1 (fresh sample), 2 (aged sample), and 3 (aged sample). The observed molecular weight from peak 1 and peak 2 are 3,873.1 Da (the theoretical molecular weight of IAPP-S20G is 3,873.3 Da). Peak 3 likely corresponds to a single deamidation for that subset of peptide (m/z shift of 0.242 Da, observed molecular weight: 3,874.1 Da). Note that different sample concentrations were injected in (A) and (B) that result in different peak widths in the chromatograms (STAR Methods). No degradation of the peptide was observed over the incubation period.

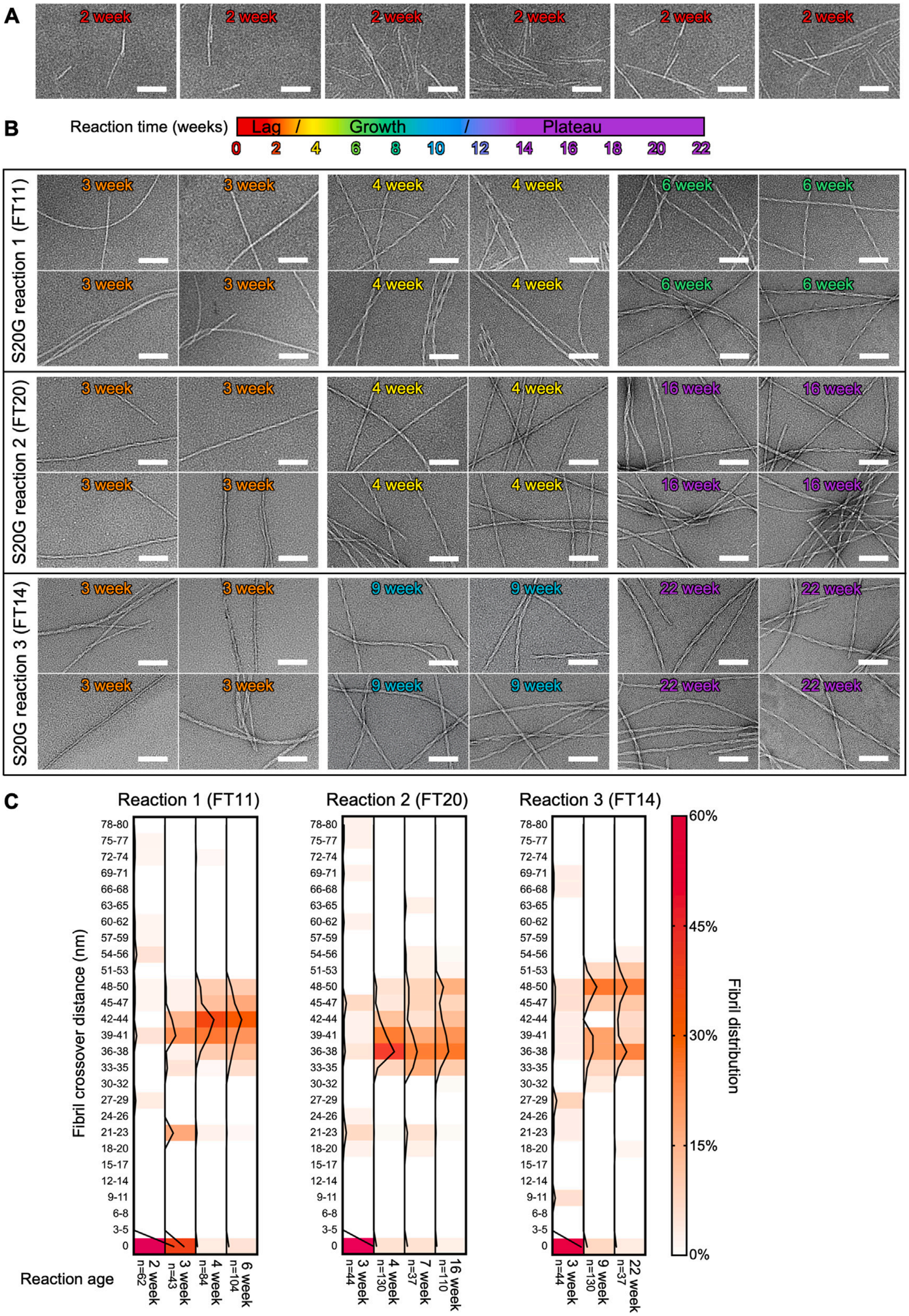

(legend on next page)

Figure S2. Negative stain EM images showing maturation of fibrils in three separate IAPP-S20G reactions, related to Figure 2

(A) Representative sections of negative stain EM images from IAPP-S20G (fibril tube 11 [FT11]) at 2 weeks showing the first fibrillar material to appear, with no oligomers evident. The scale bar (white) represents 100 nm.

(B) Four representative sections of negative stain images from different time points obtained for three separate fibrillation reactions. The color key indicates the sample age in reference to the lag, growth, and plateau phases of the amyloid assembly reaction.

(C) Heatmap plots of fibril crossover distribution separated for each reaction corresponding to the negative stain images in (B). The style and color key are similar to Figure 2C. Each fibril contributed one crossover value, with the number of fibrils measured reported on the x axis for each sample. Each reaction shows predominantly untwisted fibrils at 2–3 weeks, which in each case then matures with the majority of fibrils containing repeating crossover lengths in the range of 33–50 nm at later time points.

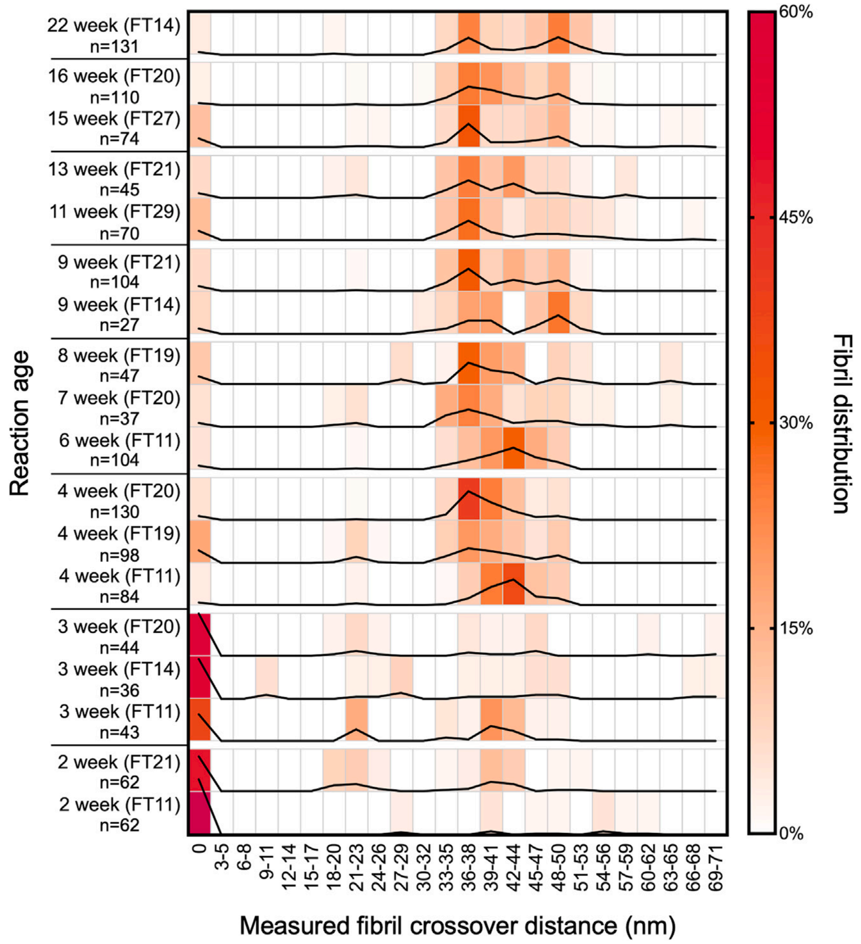

Figure S3. Comprehensive crossover distribution analysis from all of the reactions imaged by negative stain EM, related to Figure 2
Heatmap showing the percentage distribution of different fibril crossovers measured from negative stain EM images of different IAPP-S20G reactions at multiple time points. Similar style and colorings to Figure 1C. Each fibril contributed one crossover value with the number of fibrils measured reported on the y axis for each sample.

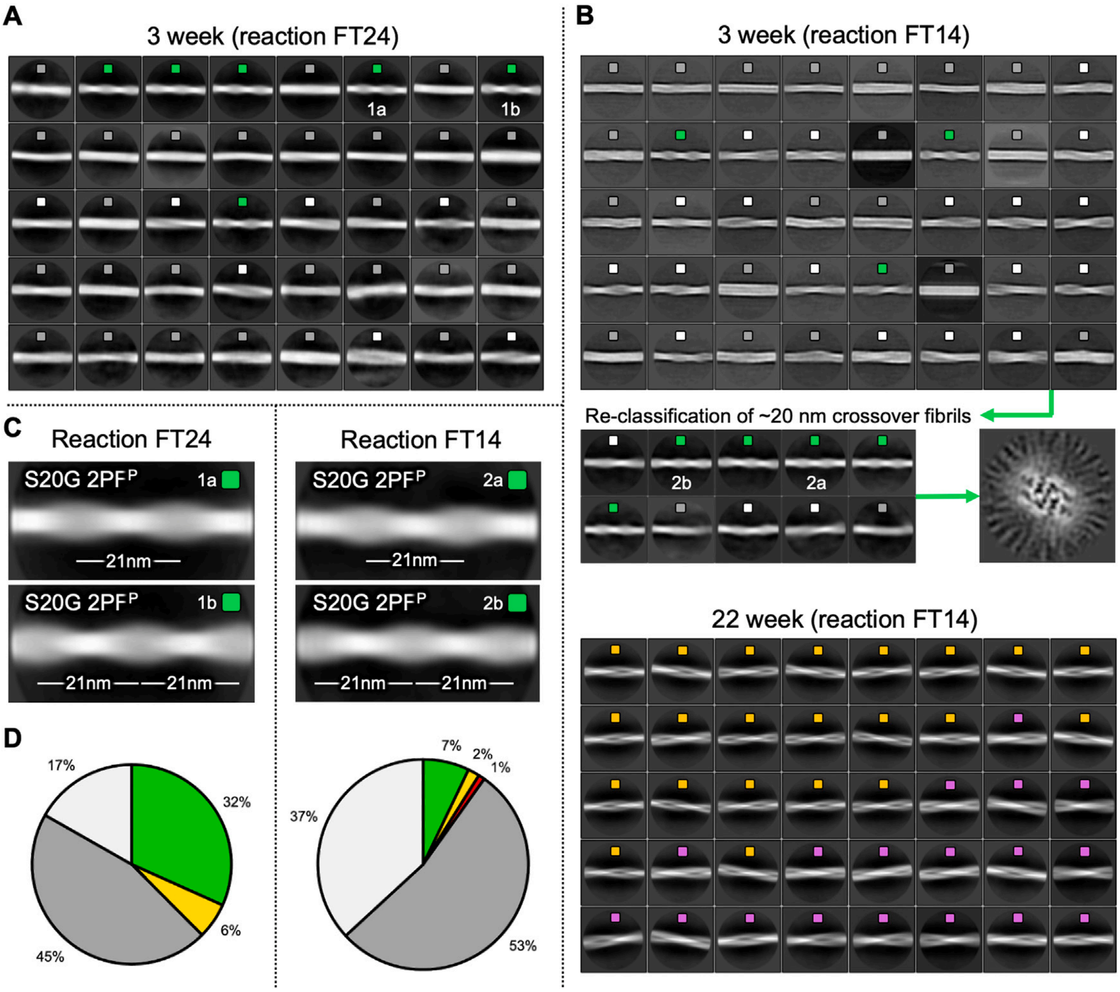

Figure S4. Cryo-EM analysis of a replicate 3-week reaction shows the same 2PF^P fibril, related to Figure 3

The 2D class averages of (A) the 3-week cryo-EM dataset from reaction FT24 as shown in Figure 3. (B) 2D class averages from the replicate 3-week cryo-EM dataset (top) and below it the 22-week cryo-EM dataset (bottom) as shown in Figure 3 (both taken from the same IAPP-S20G reaction vial, FT14). For the 3 week FT14 dataset, the initial classification was run with a larger low-pass resolution filter to help identify rare morphologies. 2D class averages from the second round of classification after selecting just the 15,085 segments in 2PF^P-like classes (green) are shown (middle) where clearer 2PF^P-like classes can be seen. Following this, 3D classification with 3,771 segments yielded a fibril core resembling that of the 2PF^P structure as judged by the displayed central slice of the map (middle, right). In all panels, the color-coding is based on the apparent fibril morphology as used in Figure 3 (gray is unfeatured, white is partially twisted but unidentifiable, green is 2PF^P, yellow is 2PF^L, and purple is 4PF^{CU}).

(C) Selected example 2D class averages from each of the replicate 3-week cryo-EM datasets showing the presence of the 2PF^P-like fibrils in each sample. The corresponding class averages used in (C) are labeled in (A) and (B) (1a, 1b, 2a, and 2b).

(D) Pie charts representing the distribution of fibril morphologies based on the displayed 2D classifications of the 3-week cryo-EM datasets, color-coded as in Figure 3.

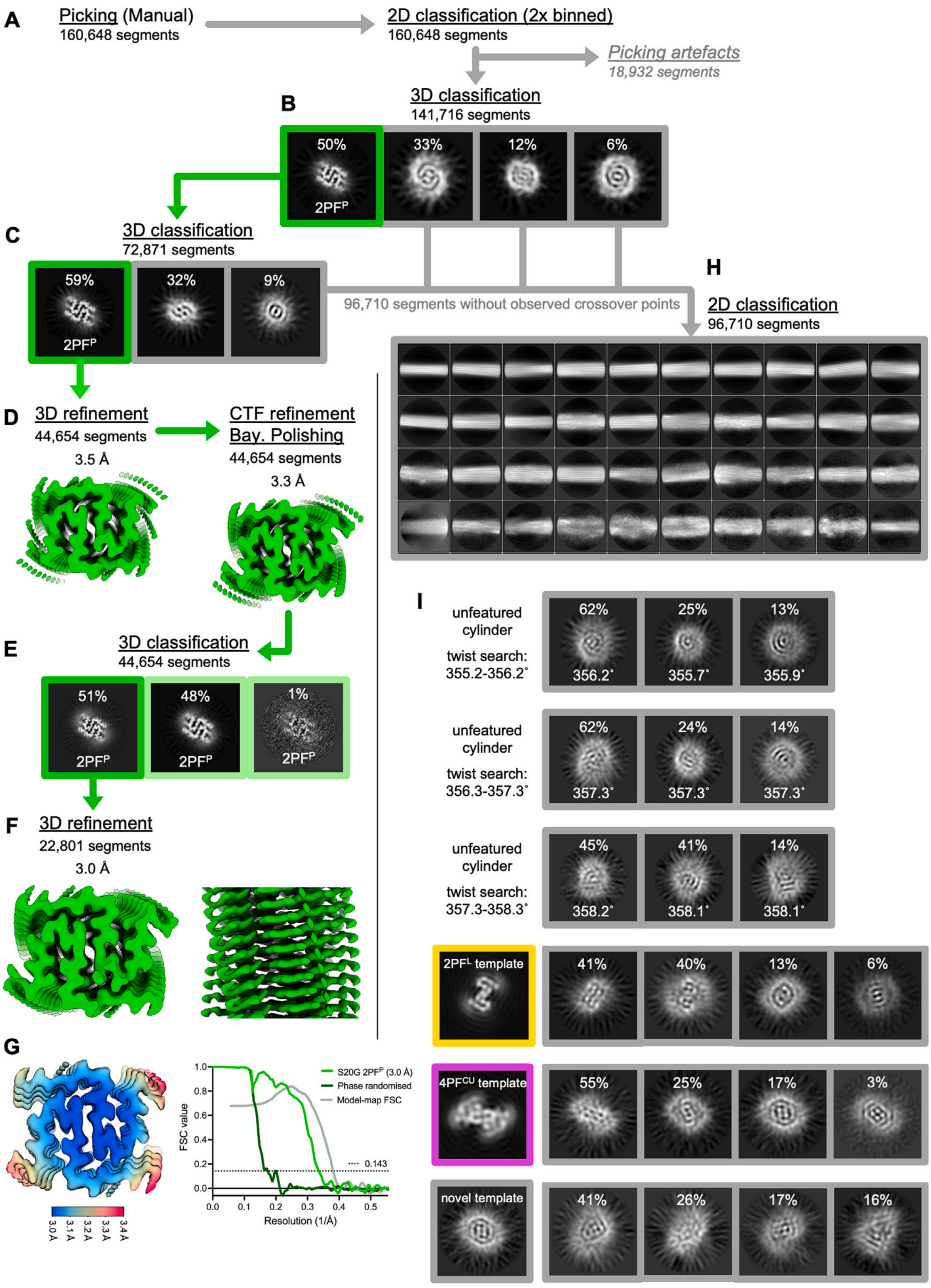

Figure S5. Processing scheme for the 3 week (lag phase) cryo-EM dataset, related to Figure 4 and STAR Methods

(A) Initial picking and 2D classification processing steps to remove picking artifacts.

(B) Slice view of the output from the first round of 3D classification with selected $2PF^P$ class highlighted in green.

(C) Output from the second round of 3D classification on the $2PF^P$ segments.

(legend continued on next page)

(D) Initial refinement outputs for 2PF^{P} before and after Bayesian polishing.
(E) Output from a final round of 3D classification without image alignment using the angular assignments from the previous refinement.
(F) The final refined 2PF^{P} map at 3.0-Å resolution, with perpendicular view showing clear separation of the fibril layers.
(G) Local resolution colored 2PF^{P} map, calculated using RELION-4.0 and the Fourier shell correlation (FSC) curves from the final refinement.
(H) The rejected classes from the first two rounds of 3D classification were combined (gray) as the pool of non-2PF^{P} fibril segments. Unbinned 2D class averages are shown from classification of the 96,710 segments, showing some internal structural elements but with limited observable/measurable crossovers.
(I) Some examples are shown from the multiple classification runs attempted on these segments with different templates (either cylindrical, other IAPP-S20G polymorphs or novel generated from 2D or 3D class averages from the data) and helical parameter searches, but no definitive fibril cores could be resolved.

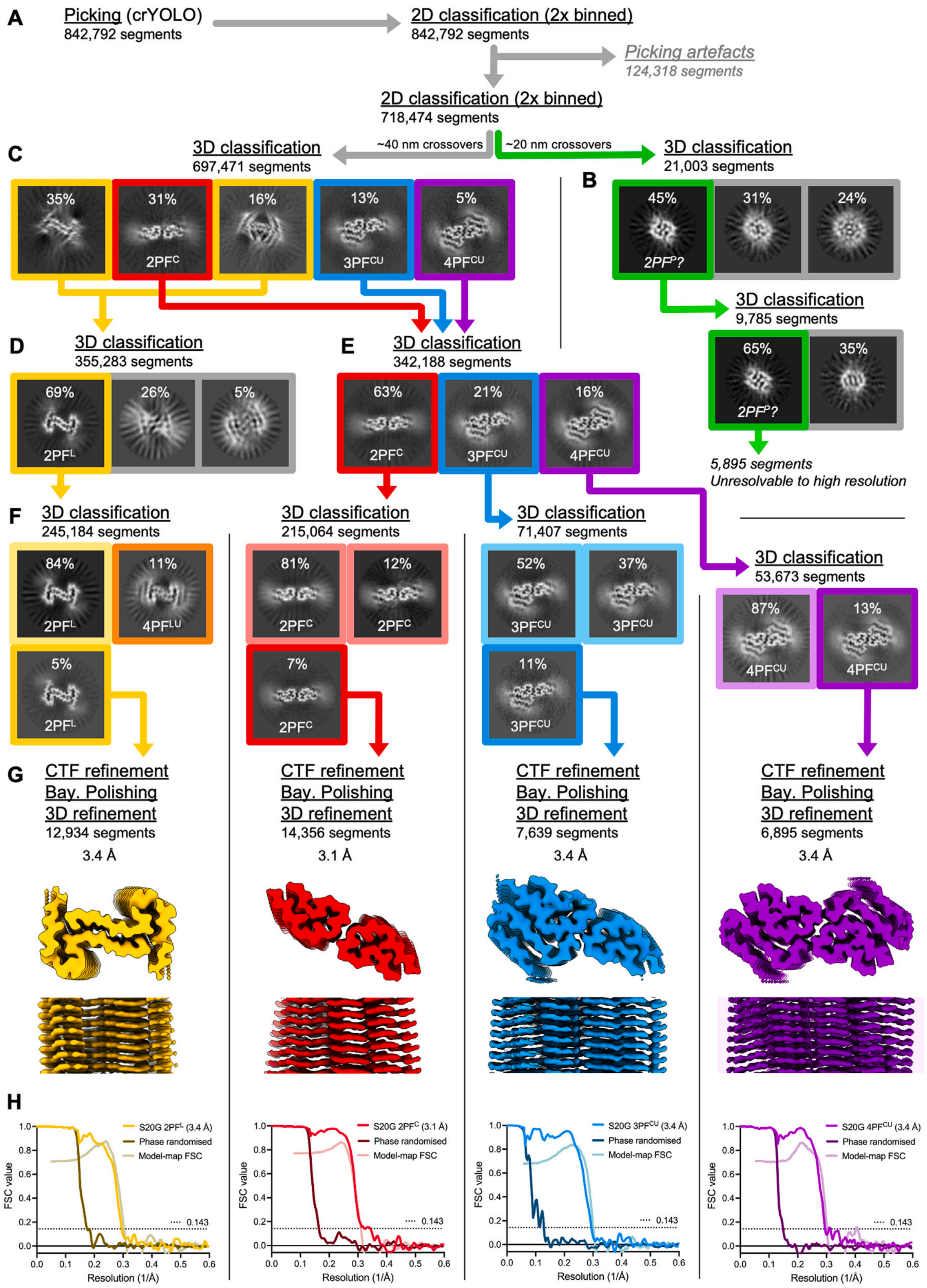

(legend on next page)

Figure S6. Processing scheme for the 6-week (growth phase) cryo-EM dataset, related to Figure 4 and STAR Methods

(A) Initial picking and 2D classification processing steps to remove picking artifacts. In the final 2D classification run, fibril segments were split into two populations: classes showing ~40-nm crossovers and those showing ~20-nm or unfeatured crossovers.

(B) Outputs from consecutive 3D classification runs with the ~20-nm-segment pool, using the $2PF^{P}$ structure as a template. A high-resolution map could not be obtained from the small population of segments.

(C) Output from the first round of 3D classification on the ~40-nm-segment pool using a $2PF^{C}$ initial model as the template. The outputs were split two polymorph pools, $2PF^{C}$/$3PF^{CU}$/$4PF^{CU}$ in one and $2PF^{L}$/ambiguous classes in the second, for further processing.

(D and E) Output from the second round of 3D classification for each of the separate subsets, from which the individual polymorphs were separated.

(F) Output from the final round of 3D classification for each separate polymorph segment pool, from which only the respective ordered classes were selected for refinement to high resolution.

(G) The final deposited maps for each solved fibril form with perpendicular views showing separation of the individual layers.

(H) FSC curves from the final refinement of each solved structure. The fibril forms are colored by polymorph using the same color scheme as in the other figures in this work.

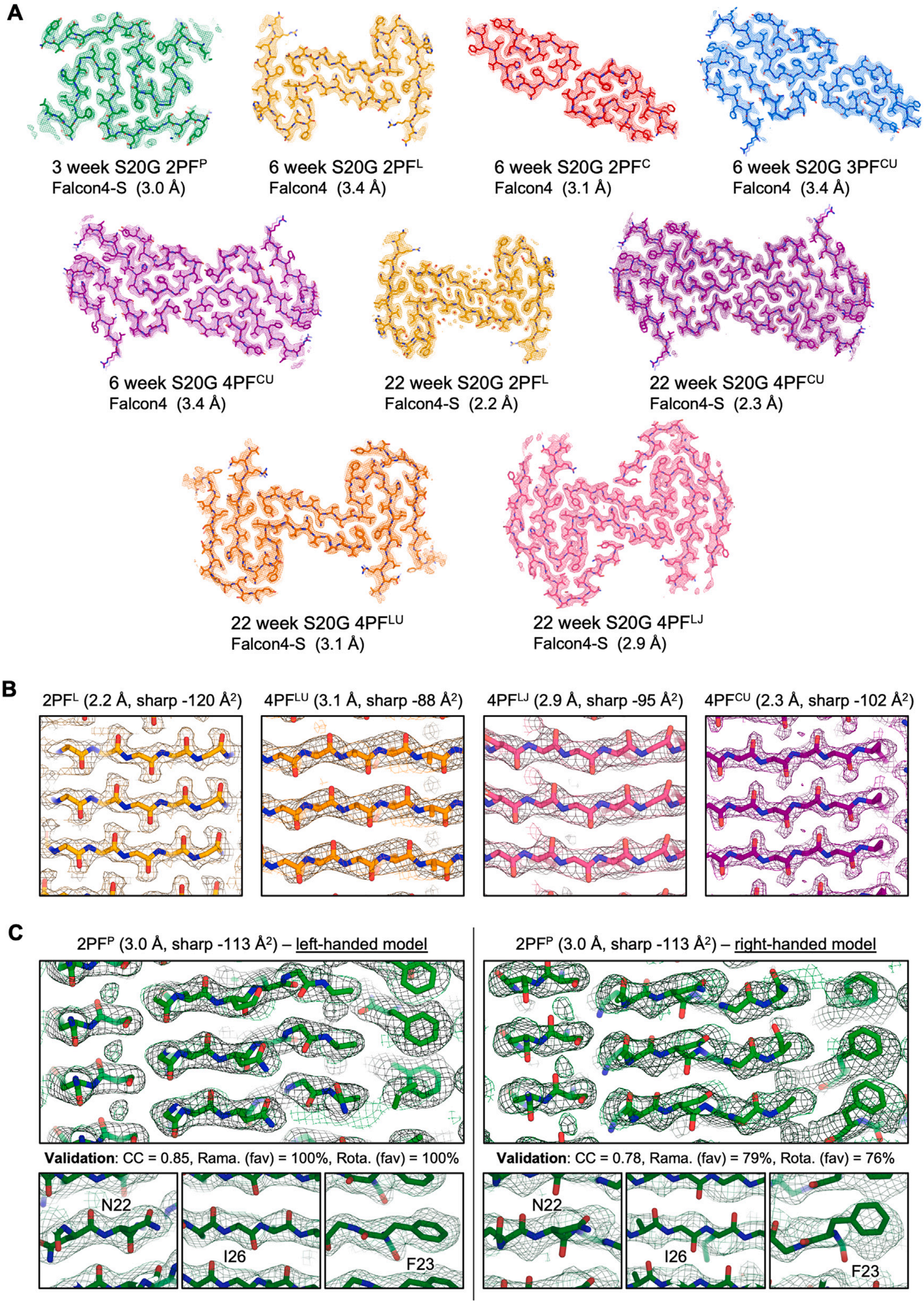

(legend on next page)

Figure S7. The quality of the cryo-EM maps facilitates modeling of left-handed fibrils for each solved IAPP-S20G fibril polymorph, related to Figure 5

(A) The nine deposited IAPP-S20G models (sticks) and associated cryo-EM maps (mesh) are displayed, colored by polymorph as in previous figures. Data were acquired using a Falcon4 or Falcon4 plus Selectris energy filer, as indicated (Falcon4 and Falcon4-S, respectively).

(B) Sections of the cryo-EM maps and fitted, left-handed models for the novel S20G $2PF^{L}$, $4PF^{LU}$, $4PF^{LJ}$, and $4PF^{CU}$ fibril structures, focusing on backbone b-strand segments. Previously published atomic force microscopy images of $2PF^{C}$ and $3PF^{CU}$ confirmed that these fibrils were left-handed.[48] The lower-resolution $4PF^{LU}$ and $4PF^{LJ}$ maps, suggest a left-handed fibril, which is validated further by the structural alignments with $2PF^{L}$ (Figure 5E), which is clearly left-handed in the displayed 2.2-Å cryo-EM map.

(C) The final novel S20G polymorph is $2PF^{P}$ for which the map at 3.0-Å resolution infers a left-handed fibril. Similar views of the structure as determined (left) and after inverting the hand of the map (right), shown with a refined left-handed or right-handed model, respectively. The backbone carbonyls, restrained by the fitting of resolved side chains, fit the density better in the left-handed model as validated by the post real-space refinement statistics (CC, correlation coefficient between the masked map and the model; Rama., favored Ramachandran orientations; Rota., favored rotamer orientations).

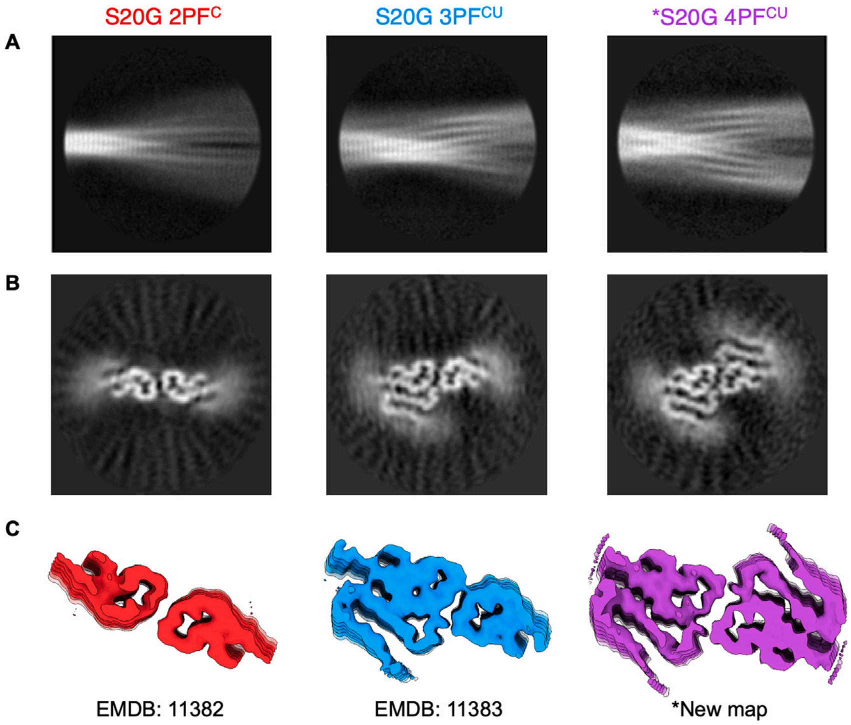

Figure S8. Re-processing of the IAPP-S20G dataset from our previous study shows the presence of the 4PF^{CU} polymorph, related to Figure 4

The IAPP-S20G cryo-EM dataset from our previous study,[48] from which the 2PF^{C} and 3PF^{CU} polymorphs were initially solved, was re-processed in the light of the findings of the current study.

(A) 2D class averages representing the 2PF^{C}, 3PF^{CU}, and the 4PF^{CU} polymorphs can be seen in the data.

(B) Central slices of the resulting maps from 3D classification for each polymorph.

(C) The previously deposited 2PF^{C} and 3PF^{CU} maps are shown, in addition to a new map solved for the 4PF^{CU} fibril polymorph (noted *New map), which represented 10% of the total population of segments.

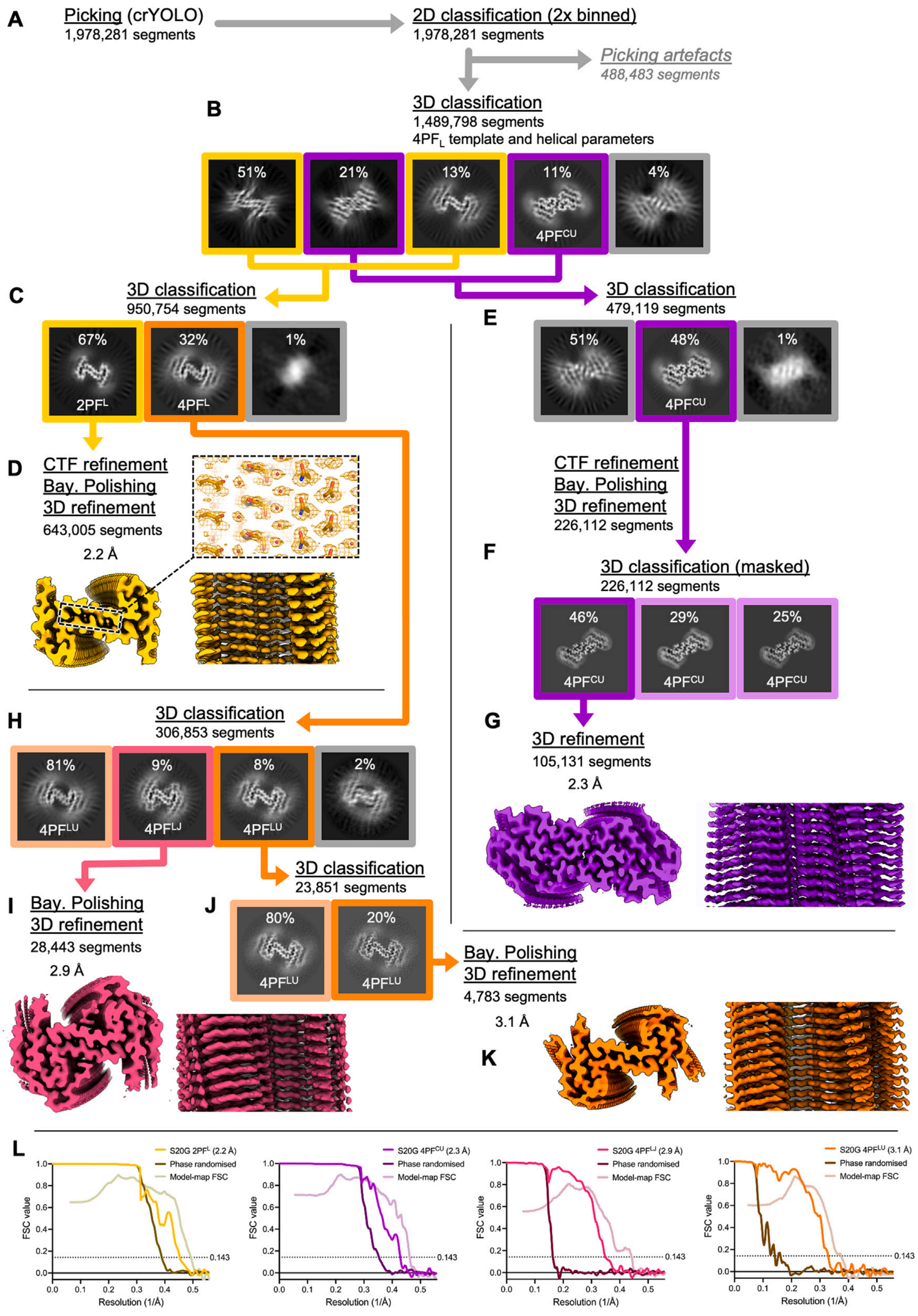

(legend on next page)

Figure S9. Processing scheme for the 22-week (plateau phase) cryo-EM dataset, related to Figure 4 and STAR Methods

(A) Initial picking and 2D classification processing steps to remove picking artifacts. In the final 2D classification run, fibril segments were split into two populations: classes showing ~40-nm crossovers and those showing ~20-nm or unfeatured crossovers.

(B, C, and E) (B) Output from the first round of 3D classification on all the fibril segments using a $4PF^{CU}$ initial model as the template. The outputs were split two polymorph pools, based on resemblance to $2PF^{L}$ or $4PF^{CU}$, respectively. Output from the second round of 3D classification for the (C) $2PF^{L}$ and the (E) $4PF^{CU}$ segment pools, respectively.

(D) The final deposited map for the solved $2PF^{L}$ fibril form with perpendicular view showing separation of the individual layers and boxed close-up view of the map (with extra sharpening applied compared with the deposited map) and model showing the ordered water channel at the inter-subunit interface.

(F) The polished, refined $4PF^{CU}$ segments were used for 3D classification without image alignment with a mask applied around the external subunits of the fibril core. An output class with more ordered density for the backbone of the external peptide chains was selected for refinement.

(G) The final deposited $4PF^{CU}$ map a with perpendicular view showing separation of the individual layers.

(H) $4PF^{L}$ segments selected from (C) were further 3D classified, revealing a mixture of two distinct fibril structures.

(I) The final deposited map for the $4PF^{LJ}$ fibril form with a perpendicular view.

(J) Output from a final round of 3D classification with the $4PF^{LU}$ segments revealed a more ordered class from which the structure could be solved to high resolution.

(K) The final deposited map for the $4PF^{LU}$ fibril form with a perpendicular view.

(L) FSC curves from the final refinement of each solved structure from the 22-week dataset. Throughout, the fibril forms are colored by polymorph as in the other figures in this work.

CellPress
OPEN ACCESS

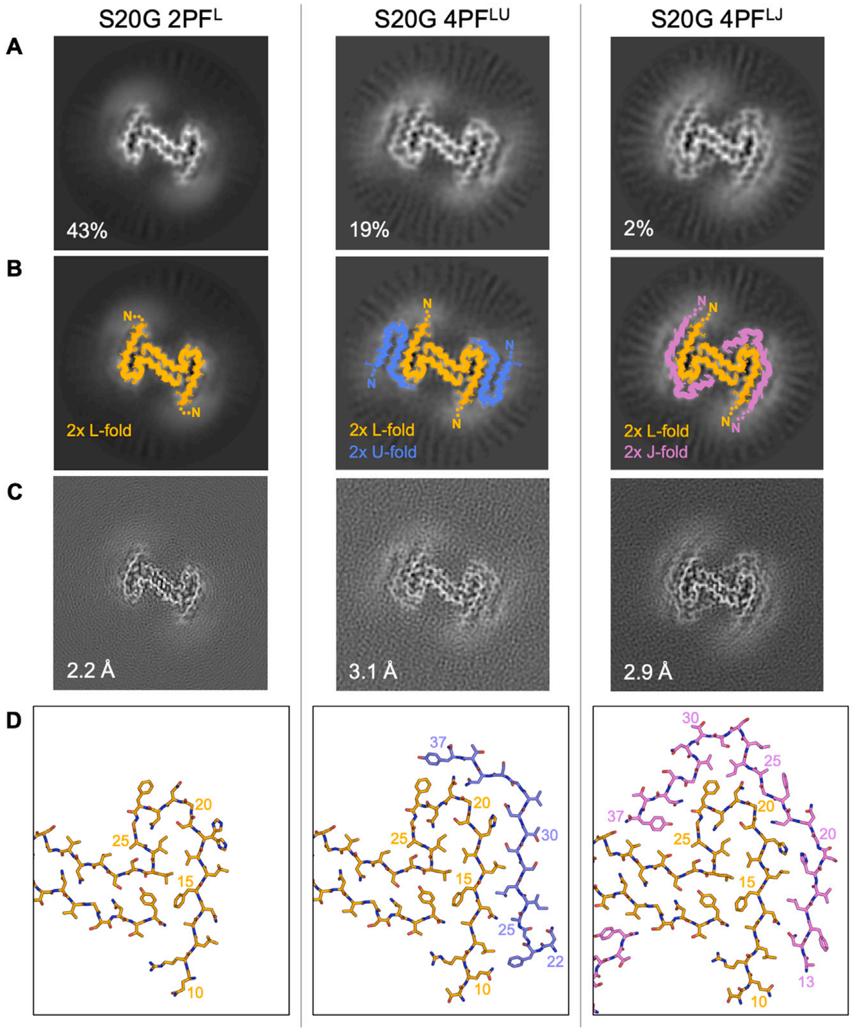

Figure S10. Summary of the structures of the L-lineage IAPP-S20G fibril assemblies, related to Figure 5

(A) Slice view of 3D classification maps for each fibril form, 2PF^{L}, 4PF^{LU}, and 4PF^{LJ} with segment distributions labeled relative to the total number of fibril segments in the 22-week dataset.

(B) The same slices from (A) are shown with colored cartoon models of the respective subunit folds (generated from the solved PDB models in PyMol) overlaid to highlight the different IAPP-S20G folds in each assembly.

(C) Slices of the final deposited maps for each fibril form, with final resolution labeled.

(D) View of the final PDB models for each fibril form, focusing on the interaction surface for one of the external subunits. In this figure, the models are colored by subunit fold as in Figure 5.

A

	WT $2PF^{S}$	S20G $2PF^{P}$	S20G $2PF^{C}$	S20G $2PF^{L}$	S20G $3PF^{CU}$	S20G $4PF^{CU}$	S20G $4PF^{LU}$	S20G $4PF^{LJ}$
**Composition**								
Residues ordered (per chain)	13-37	12-37	13-37	10-37	11-37, 11-37	11-37, 11-37	10-37, [a]11-37	9-37, 13-37
Protofilaments	2 (2S)	2 (2P)	2 (2C)	2 (2L)	3 (2C, 1U)	4 (2C, 2U)	4 (2L, 2U)	4 (2L, 2J)
**FoldX $\Delta G^{o fold}$ (kcal/mol)**								
Per fibril layer	-65.5	-47.0	-47.9	-57.6	-79.3	-111.9	-128.1	-122.4
Per residue	-1.31	-0.90	-0.98	-1.03	-0.98	-1.04	-1.16	-1.13
**Eisenberg ΔG^{o} (kcal/mol)**								
Per fibril layer	-26.0	-32.4	-28.3	-30.6	-45.8	-60.8	-60.3	-60.9
Per residue	-0.52	-0.62	-0.57	-0.55	-0.58	-0.56	-0.54	-0.56
**Average ΔG^{o} (kcal/mol)**								
Per fibril layer	-45.8	-39.7	-38.1	-44.2	-62.6	-86.3	-94.2	-91.7
Per residue	-0.92	-0.76	-0.77	-0.79	-0.8	-0.8	-0.85	-0.85

B

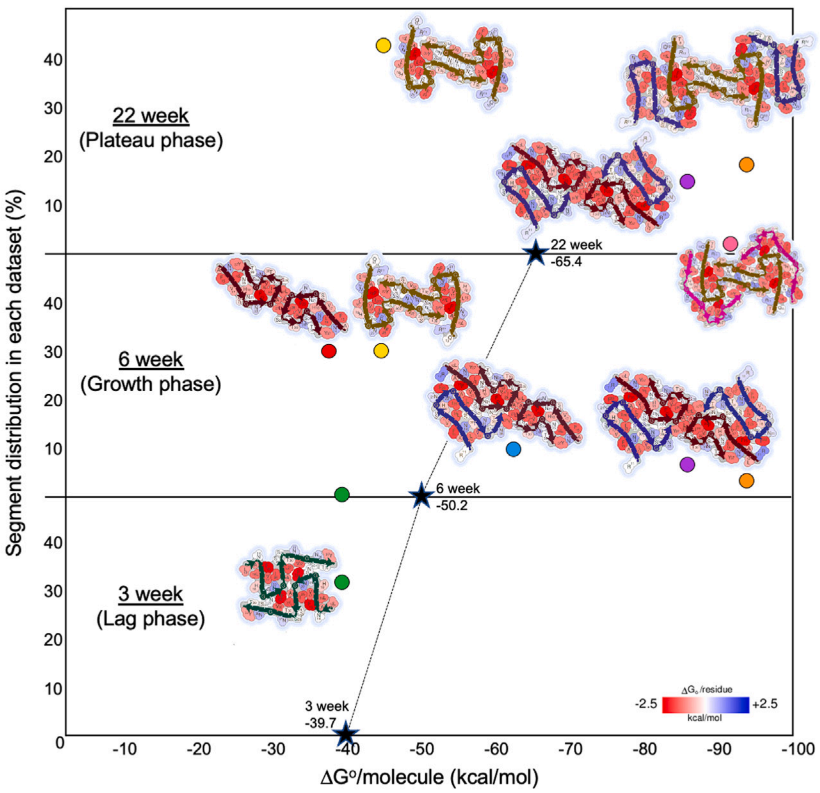

Figure S11. Larger potentially more stable fibril assemblies accumulate as the reaction progresses, related to Figure 6

(A) Computed free energy stability values for each polymorph, calculated as described in STAR Methods using the FoldX[55] or Eisenberg/Sawaya[12] methods. Values per fibril layer represent the sum change in ΔG for folding for a single, middle layer of each fibril model, i.e., for two subunits of all 2PF structures, three for 3PF, and four for 4PF structures. Values per residue represent the average change in ΔG per residue in the fibril layer, i.e., the per fibril layer value divided by the number of ordered residues. The average ΔG section represents averaged values from the two methods for each case.

(B) Stacked summary chart showing the percentage distribution of segments from cryo-EM processing vs. the average calculated ΔG^{o}/molecule (for one fibril layer) of each polymorph in each dataset from (A). The position of each structure is indicated by the filled circles, colored by polymorph as in (A). The weighted average ΔG^{o}/molecule within each dataset is indicated by a black star with a dotted line tracking its progression between the time points. Eisenberg/Sawaya[12] ΔG^{o}/residue coloring of each solved structure is shown near its respective location in the chart. The images were generated using scripts provided by Michael Sawaya as used for the webserver Amyloid Atlas[12] and are colored using the scale indicated in the bottom right.

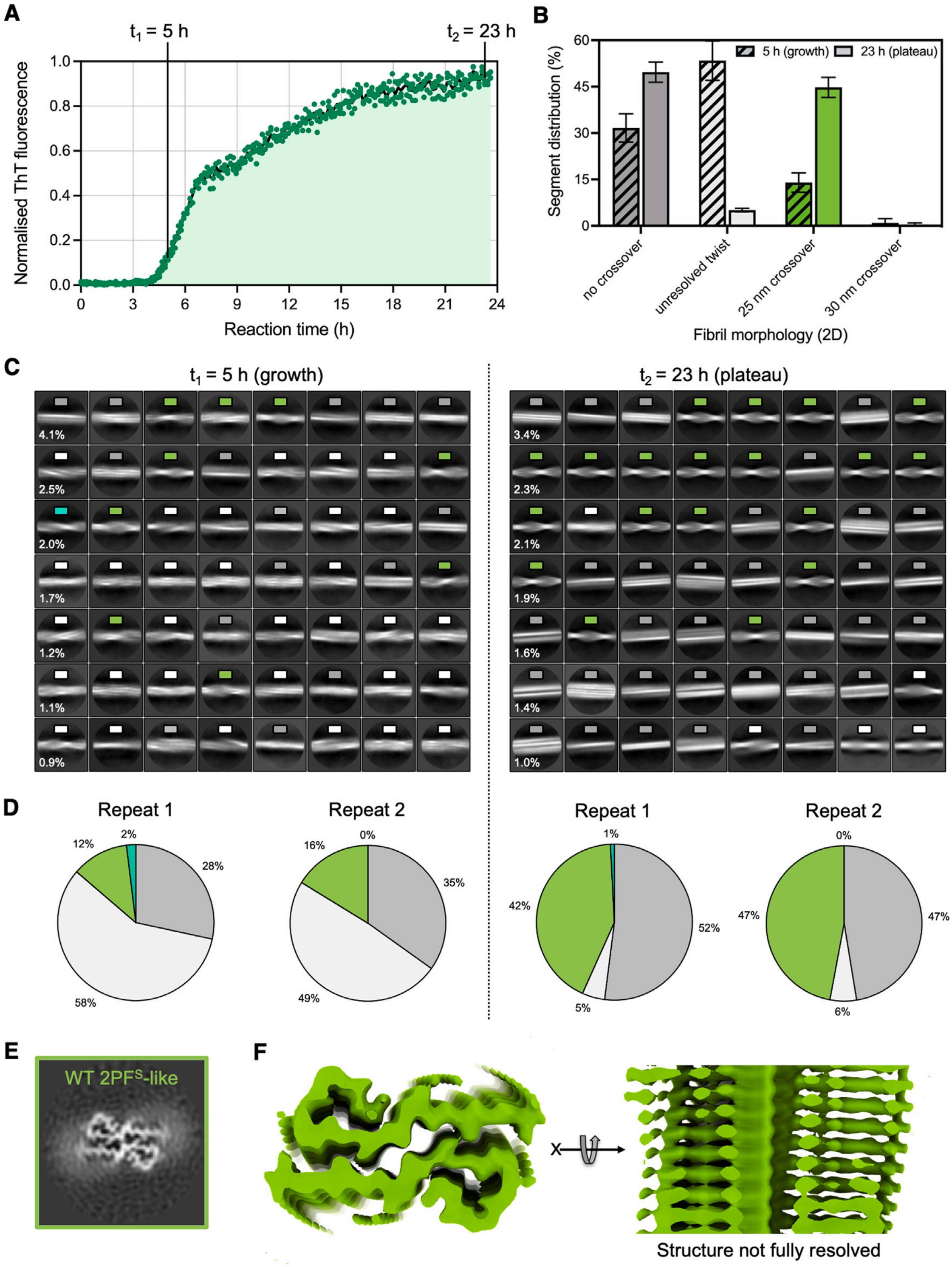

Figure S12. Time course of WT hIAPP amyloid fibrils formed *in vitro* in 96-well plates showing a subtle maturation of fibril polymorphs, related to Figure 3

(A) ThT plot with three replicate reporter reactions continuously sampled over time in a plate reader, grown quiescently in the presence of 10 μM ThT (STAR Methods). Replicates are shown as circles with a line plot tracking the average value at each time point. The two time points (5 and 23 h) sampled by cryo-EM datasets, from parallel reactions taken simultaneously, but in the absence of ThT, are labeled on the plot.

(legend continued on next page)

(B) Summary bar chart of the different polymorph distributions observed from cryo-EM 2D class averages from reactions sampled at 5 h (diagonally divided bars) and 23 h (solid filled bars). Each value is the average percentage from two replicate reactions, with the error bars shown in black. Different replicate reactions were used for all four datasets as the entire contents of a well were harvested at each time point.
(C) The most populated 2D class averages for each reaction are shown for the 5 h (left) and 23 h (right) datasets. The class averages are color coded according to observed morphology, as in the bar chart (B). The box size of each class average is ∼50 nm.
(D) Pie charts summarizing the polymorph distribution from each separate dataset displayed in (C), colored as in the previous panels.
(E) Central slice (calculated by averaging the central six voxel-layers to represent a single helical layer) of a map from the only fibril morphology that could be resolved in 3D classification and refinement. This 25-nm crossover polymorph closely resembles the patient-seeded structure TW2 from Cao et al.[30] (PDB: 7M62), including the orientation of the C-terminal Y37 residue, and differs significantly from previous structures of WT IAPP fibrils (2PF^{S}) obtained previously under different growth conditions.[30,48,50]
(F) Perpendicular views of the refined cryo-EM map show detailed side chain densities, but a failure to completely resolve the inter-strand helical layers in this dataset means that the structure cannot be refined further.

CellPress
OPEN ACCESS

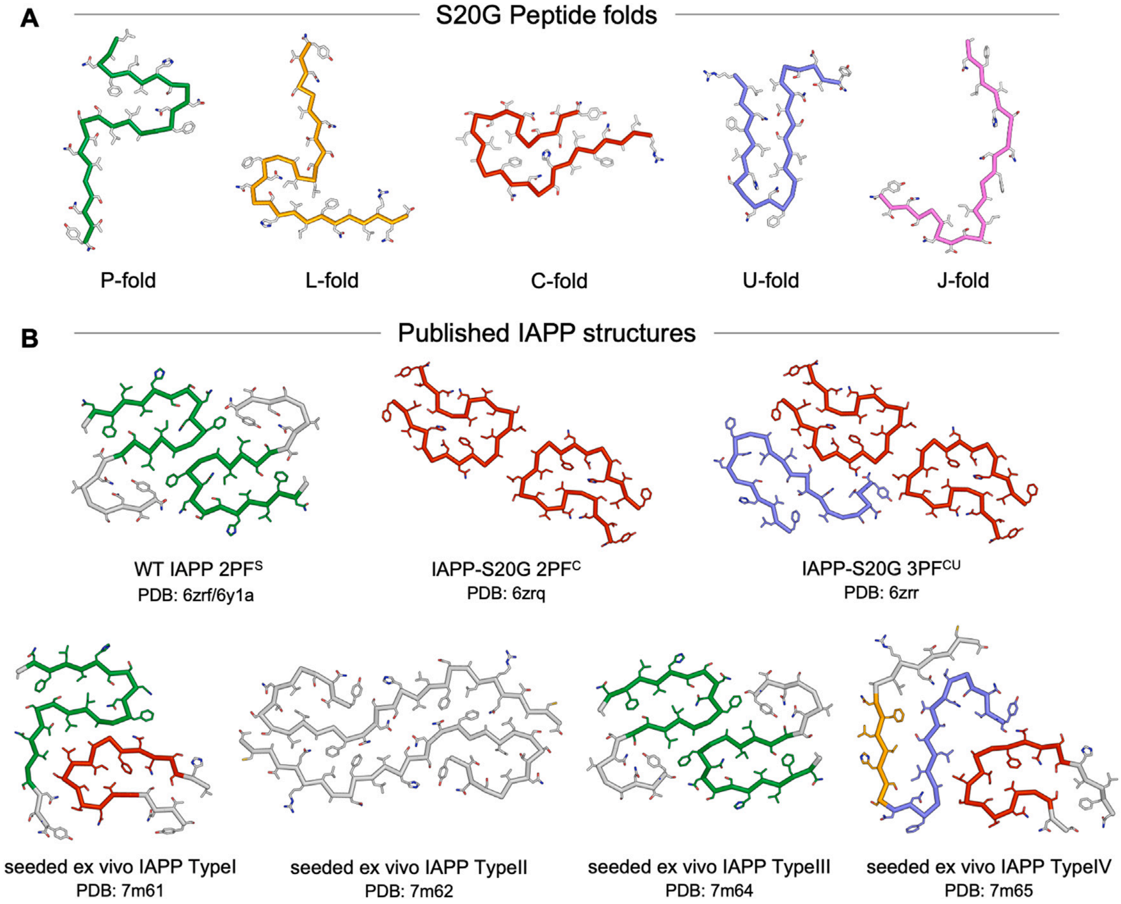

Figure S13. Summary of the discovered IAPP-S20G subunit folds and their relation to other published IAPP fibril structures, related to Figure 5

(A) Cartoon view of each of the five unique IAPP-S20G subunit folds found within the fibril structures solved in this work.

(B) Previously published IAPP fibril structures[30,48,50] with regions matching to the folds in (A) colored accordingly.
